# A bird’s-eye view for future ionic thermoelectrics: from ions to products

**DOI:** 10.1093/nsr/nwag283

**Published:** 2026-05-14

**Authors:** Ziyao Xu, Mao Yu, Qikai Li, Chunjing Wang, Yuxiu Yao, Ruixin Wei, Cheng Chi, Cheng Yan, Xiaogang Zhang, Yinghong Xu, Baoling Huang, Dongyan Xu, Yun Shen, Zehao Zhao, Shien-Ping Feng, Weishu Liu, Hulin Zhang, Shangchao Lin

**Affiliations:** Institute of Engineering Thermophysics, School of Mechanical Engineering, Shanghai Jiao Tong University, Shanghai 200240, China; Department of Materials Science and Engineering, Southern University of Science and Technology, Shenzhen 518055, China; Department of Materials Science and Engineering, Southern University of Science and Technology, Shenzhen 518055, China; Department of Systems Engineering, City University of Hong Kong, Hong Kong 999077, China; College of Integrated Circuits, Taiyuan University of Technology, Taiyuan 030024, China; College of Integrated Circuits, Taiyuan University of Technology, Taiyuan 030024, China; College of Integrated Circuits, Taiyuan University of Technology, Taiyuan 030024, China; Key Laboratory of Power Station Energy Transfer Conversion and System of Ministry of Education, School of Energy Power and Mechanical Engineering, North China Electric Power University, Beijing 102206, China; School of Mechanical, Medical and Process Engineering, Faculty of Engineering, Queensland University of Technology, Brisbane 4000, Australia; College of Materials Science and Technology, Nanjing University of Aeronautics and Astronautics, Nanjing 211106, China; College of Materials Science and Technology, Nanjing University of Aeronautics and Astronautics, Nanjing 211106, China; Department of Mechanical and Aerospace Engineering, The Hong Kong University of Science and Technology, Hong Kong 999077, China; Department of Mechanical and Automation Engineering, The Chinese University of Hong Kong, Hong Kong 999077, China; Department of Mechanical and Automation Engineering, The Chinese University of Hong Kong, Hong Kong 999077, China; Department of Mechanical and Automation Engineering, The Chinese University of Hong Kong, Hong Kong 999077, China; Department of Systems Engineering, City University of Hong Kong, Hong Kong 999077, China; Department of Materials Science and Engineering, Southern University of Science and Technology, Shenzhen 518055, China; College of Integrated Circuits, Taiyuan University of Technology, Taiyuan 030024, China; Institute of Engineering Thermophysics, School of Mechanical Engineering, Shanghai Jiao Tong University, Shanghai 200240, China

**Keywords:** ionic thermoelectrics, thermodiffusion effect, thermogalvanic effect, low-grade heat harvesting, energy conversion and storage, multifunctional device

## Abstract

The ionic thermoelectric (i-TE) technology offers a compelling pathway for harvesting low-grade heat, distinguished by its exceptionally high thermopower and inherent material versatility. However, development in this field is constrained by the complex interplay among electrochemical, thermodynamic, and transport phenomena, which poses significant challenges to the fundamental understanding, accurate performance evaluation, and systematic screening of new materials. This review provides a systematic overview and outlook of the i-TE landscape, bridging fundamental principles with future applications. We begin by deconstructing the core components—electrolytes and electrodes—to elucidate the material design strategies that govern the performance. The discussion then progresses to a multi-scale evaluation of key metrics, from intrinsic i-TE properties to device-level energy conversion and storage capabilities. A central focus is placed on dissecting the persistent chemical and physical challenges, including ion selectivity, transport dynamics, and interfacial engineering. This review further surveys the emerging applications of i-TE, ranging from wearable power generation and active cooling to multimodal sensing and integrated multifunctional systems. Furthermore, we highlight the paradigm-shifting potential of synergistic systems, where coupling thermoelectric effects with electrochemical, photocatalytic, or hydrovoltaic processes unlocks unprecedented functionalities and performance enhancements. Ultimately, this review synthesizes current understanding to propose a strategic roadmap for this field. It outlines the key scientific and engineering perspectives on standardization, scalable manufacturing, and reliability that are essential to translate laboratory innovations into viable commercial technologies.

## Teaser

This review spans ionic thermoelectrics from historical roots and fundamental concepts to key chemical and physical challenges, highlights their versatile capabilities in energy conversion, storage, cooling, and sensing, and charts a roadmap toward commercialization.

## INTRODUCTION

Ionic thermoelectrics (i-TEs) offer an alternative approach for energy conversion between heat and electricity, utilizing ions as the energy carrier, in contrast to thermoelectric semiconductors [1,2] or organic thermoelectrics [3] that rely on electrons or holes (electronic thermoelectrics, e-TEs). One of the unique advantages of i-TEs is their high thermopowers, that is, *S*_i_  $= - \frac{{{V}_{\mathrm{H}} - {V}_{\mathrm{C}}}}{{{T}_{\mathrm{H}} - {T}_{\mathrm{C}}}}$, where ${V}_{\mathrm{H}}$ and ${V}_{\mathrm{C}}$ are the thermovoltage of the hot electrode at temperature ${T}_{\mathrm{H}}$ and the cold electrode at temperature ${T}_{\mathrm{C}}$, respectively [4]. The definition of thermopower [5] is ostensibly the same as the term ‘thermopower’ [6,7] utilized in the classic e-TE community, but more general for i-TE materials [8]. Here, we refer to thermopower as an intrinsic physical parameter that describes i-TE materials in all condensed phases, including molten salts, solid electrolytes, solutions, and gels, and quantifies their ionic thermal-electrical conversion under temperature differences. The connotation of this definition excludes the effect of the electrode impact and environmental ingredients to simplify the thermodynamic description of i-TE phenomena. As a power source, the i-TE systems may have no obvious advantage over the traditional e-TE counterparts in terms of the output power density. However, the stretchability, multifunctionality, and solution-processability of gels afford new opportunities for the i-TEs in the fields of wearable electronics [9,10]. Beyond their high thermopower, i-TE systems offer additional advantages through versatile working modes, such as hybrid devices integrating conversion and storage, sensing, low-grade heat energy harvesting and synergistic combinations with other energy-conversion mechanisms [11–14]. Furthermore, i-TE materials also raise new fundamental questions related to ionic transport and charge varying, attracting increasing attention from worldwide researchers with very diverse backgrounds.

The discovery of i-TE phenomena dates back to the early 19th century. In the 1830s, Michael Faraday observed electrical conduction in liquids [15] and voltage generation from electrode temperature gradients [16], which was theoretically explained in 1889 by Walther Nernst [17]. The early works of Faraday and Nernst laid the foundation for the thermal-electrochemical effect, also known as the thermogalvanic effect. In 1905, Nernst and Merriam formalized the diffusion-based theory of electrode processes[18], which helped reinforce the theoretical basis of thermogalvanic systems. Earlier still, M. E. Bouty (1880) had demonstrated the linear relationship between thermoelectromotive force and temperature difference [19], and A. Gockel (1885) had identified Peltier-like cooling effects [20]. Early investigations into molten and solid salts, such as those by von H. Reinhold in 1928 [21] and the Lewis Research Center in 1964 [22], were hindered by mass transfer challenges. A key advancement occurred in 1976, when Brian Burrows introduced the [Fe(CN)_6_]^4−/3−^ redox couple to help clarify electrode contributions [23]. Theoretically, ionic thermodiffusion contributes to the thermoelectric potential in early thermogalvanic cells (TGCs, or thermocells), although this effect is typically regarded as negligible [24]. The temperature-derived salt concentration difference in a dilute solution within a vertical tube was identified by Charles Soret in 1879, which is also known as the Soret effect in thermodiffusion cells (TDCs) [25]. In the following years, theoretical efforts have continuously added new insights into the thermodiffusion mechanism, benefiting from the pioneering works by Eastman’s heat of transfer (${Q}^{\mathrm{*}}$) [26–28], Onsager’s irreversible thermodynamics [29], de Groot’s expression for the thermopotential [30], and Li’s hydrodynamic approach for electrolyte solutions [31]. Following a step in the 2000s, there are a few interests to revisit this puzzle regarding the ion thermodiffusion contribution to the thermoelectric voltage in polyethylene oxide (PEO) films [32] and synthetic collagen-based gels [33]. A turning point was marked by the report of exceptionally high values, reaching 7 mV K^−1^, in solutions of tetraalkylammonium ions in organic solvents [34]. Moreover, X. Crispin *et al*. [35] achieved notable advances in thermopower, reporting a breakthrough in a polyelectrolyte poly(3,4-ethylenedioxythiophene): poly(styrene sulfonate) (PEDOT:PSS)-PSSNa in 2015, followed by a record-high thermopower value exceeding 10 mV K^−1^ in a PEO-NaOH solution in 2016 [36].

**Figure 1.**
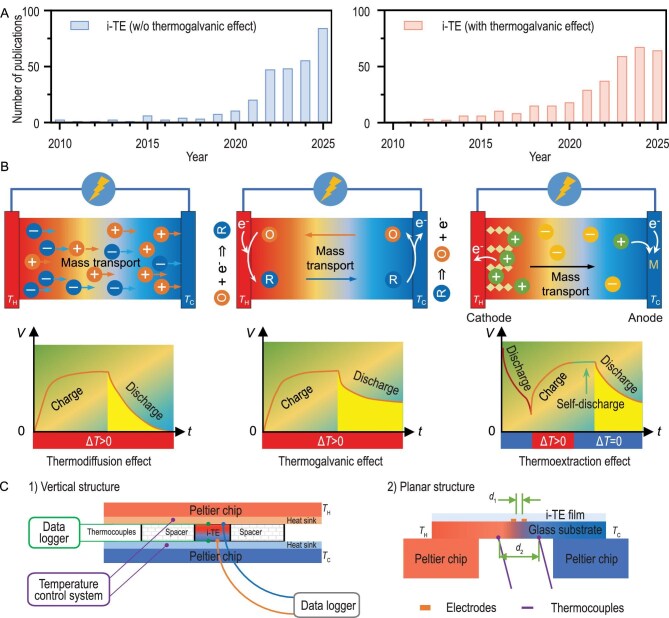
Fundamentals, measurement apparatus and working modes of i-TEs. (A) Number of publications including i-TE (without thermogalvanic effect) and i-TE (with thermogalvanic effect) materials (recorded from 2010 to 2025). Data collected from Clarivate Web of Science. (B) Schematics of ionic thermodiffusion, thermogalvanic, and thermoextraction effects under temperature difference, with the corresponding typical charge–discharge profiles depicted in the lower panel. The shadowed areas in (B) denote the electric work output during discharge. (C) Thermopower measurement apparatuses (vertical and planar structures) for i-TE devices.

Concurrently, J. Zhou’s group pioneered the development of flexible i-TE gels [37]. In 2020, the synergistic effects between thermodiffusion and thermogalvanic mechanisms [4], alongside thermosensitive crystallization-boosted liquid TGCs [38], marked a significant milestone, accelerating the development of novel i-TE materials and devices and also attracting broad attention [26–31]. Figure 1A demonstrates a sustained increase in i-TE publications since 2010, coupled with a progressive diversification of their applications.

In this comprehensive review, we provide a holistic overview of the i-TE field, bridging its deep historical roots with its recent resurgence from fundamental principles to practical applications. We begin by introducing the essential thermoelectric and mechanical properties, followed by a categorization of core electrolyte components. Furthermore, we detail device architectures, operating modes, and key performance metrics. Subsequently, we outline material design principles governing performance and critically analyze the primary physicochemical challenges facing the field. We then examine emerging applications of i-TE technology, including power generation, cooling, sensing, and other innovative avenues. Finally, we synthesize these findings to propose a strategic roadmap prioritizing standardization, scalable manufacturing, and reliability to facilitate commercial adoption.

## FUNDAMENTALS OF I-TE MATERIALS AND DEVICES

### I-TE materials

#### I-TE effects

I-TE systems consist of an electrolyte and two electrodes connected through an external circuit. A clear understanding of the energy conversion modes between functional electrodes and electrolytes is essential for advancing i-TEs toward practical applications. Depending on the ionic processes involved, thermoelectrochemical energy conversion can be classified into three categories: thermodiffusion (TD), thermogalvanic (TG), and thermoextraction (TEx) effects. The synergy among these mechanisms is also of great significance for designing high-performance i-TE devices. The schematic diagrams illustrating the underlying mechanisms of the TD, TG, and the emerging TEx effects under a temperature gradient are presented in Fig. 1B.

#### Thermodiffusion effect

The key performance of i-TEs relies on the efficient transport of ions within the material. Active thermodiffusing ions, serving as carriers in i-TE materials, directly generate thermoelectric voltage in i-TE devices through their thermodiffusive behavior (thermodiffusion effect or Soret effect) under temperature gradients. A fundamental understanding of the physical transport mechanism of ions in i-TE materials is the decisive foundation for finding strategies to optimize thermopower. The thermodiffusion thermopower of ion drift can be derived based on Onsager transport theory and is as follows [4]:


(1) \begin{eqnarray*}
{S}_{{\mathrm{TD}}} = \frac{{{{\hat{S}}}_ + {D}_ + - {{\hat{S}}}_ - {D}_ - }}{{e\left( {{D}_ + + {D}_ - } \right)}},{\mathrm{\ }}
\end{eqnarray*}

where the subscript ‘+’ or ‘−’ denotes the ion species, *e* is the elementary charge, and *D* and $\hat{S}$ are the diffusion coefficient and the Eastman entropy of transfer, respectively. From a thermodynamic perspective, $\hat{S}$ encapsulates the net heat transport associated with the restructuring of solvation shells during ionic migration, a process fundamentally governed by the temperature dependence of the Gibbs free enthalpy ($dG/dT$) [39]. Specifically, as an ion traverses from a hot region to a cold region, the energetic requirement for displacing or reorganizing its surrounding solvent molecules dictates the magnitude of the thermophoretic force; thus, a substantial entropic exchange (high $| {{{\hat{S}}}_{\mathrm{i}}} |$) directly translates into an enhanced ${S}_{{\mathrm{TD}}}$. Complementing this thermodynamic view, the kinetic sensitivity is represented by the thermodiffusion coefficient (${D}_T$), which is implicitly coupled to the entropy flux via the Soret relationship (${s}_{\mathrm{T}} = {D}_{\mathrm{T}}/D = \hat{S}/{k}_{\mathrm{B}}T$). In practice, these intricate transport characteristics not only dictate the fundamental thermopower and conductivity of i-TE materials but also underpin the overall energy conversion efficiency and commercial viability. Equation 1 relies on the classical Poisson–Nernst–Planck (PNP) framework, which often fails in complex i-TE systems by neglecting short-range correlations and nanoconfinement effects. For systems containing mobile species such as polyethylene glycol (PEG), the thermoelectric ratchet effect [40] offers a superior explanation; here, a temperature gradient rectifies activated ion hopping between adjacent sites, transcending the simplified assumptions of continuous diffusion. Consequently, elucidating the fundamental ion transport mechanisms in i-TE systems, particularly through tailoring ion-solvent/polymer interactions, selection of ions, and polymer network design, is pivotal for advancing this field.

#### Thermogalvanic effect

The i-TE materials with thermodiffusion could have high thermopower, but still struggle with low energy output. Conversely, systems employing redox couples facilitate energy conversion via simultaneous electrochemical oxidation and reduction at opposing electrodes. The internal mass convention of redox couples could make the corresponding i-TE device operate continuously especially in the liquid solvent. The choice of redox couples is central to optimizing the power output. According to the thermodynamics of electrochemical reactions [41], redox couples with large reaction entropy (${\mathrm{\Delta }}S$) yield higher output voltages when subjected to a temperature gradient. The thermopower in TGCs (${S}_{{\mathrm{TG}}}$) for the generic reaction ${{\mathrm{A}}}_{{\mathrm{ox}}} + n{e}^ - \leftrightharpoons {{\mathrm{B}}}_{{\mathrm{red}}}$, as derived from the Nernst equation, provides the theoretical underpinning for voltage enhancement [42]:


(2) \begin{eqnarray*}
{S}_{{\mathrm{TG}}} &=& - \frac{{{\mathrm{\Delta }}S}}{{nF}} - \frac{R}{{nF\left( {{T}_{\mathrm{H}} - {T}_{\mathrm{C}}} \right)}} \\
&&\times \left[ {{T}_{\mathrm{H}}\ln \frac{{{{\left( {{C}_{{\mathrm{ox}},{\mathrm{H}}}} \right)}}^{\mathrm{A}}}}{{{{\left( {{C}_{{\mathrm{red}},{\mathrm{H}}}} \right)}}^{\mathrm{B}}}} - {T}_{\mathrm{C}}\ln \frac{{{{\left( {{C}_{{\mathrm{ox}},{\mathrm{C}}}} \right)}}^{\mathrm{A}}}}{{{{\left( {{C}_{{\mathrm{red}},{\mathrm{C}}}} \right)}}^{\mathrm{B}}}}} \right].\quad\\
\end{eqnarray*}

The first term (${\mathrm{\Delta }}S/nF$) captures the reaction entropy change, which reflects the change in molecular disorder when a redox species gains or loses an electron. Here, ${\mathrm{\Delta }}S$ encompasses both the entropy contributions of covalent ligand bonding [43] and non-covalent solvation interactions [44]. A larger entropy difference between the oxidized and reduced states (for example, due to dramatic solvation shell restructuring or spin-state transitions) leads to a more significant potential shift with temperature. The second term represents the concentration effect, which quantifies how the local availability of reactants at the hot and cold electrodes further modulates the output voltage. Achieving high current and power densities requires that redox couples possess excellent chemical stability in both oxidation states, rapid redox kinetics, high reversibility, and substantial solubility in the electrolyte [45].

#### Thermoextraction effect

For i-TE systems based exclusively on redox couples, the direct conversion of a temperature gradient into electrical power is governed by TG and TEx effects, as illustrated in the middle and right panels of Fig. 1B [41,46,47]. Unlike the TG effect, where redox couples undergo redox reactions on the surface of inert electrodes, the electrodes involved in the TEx effect based i-TE device are active electrodes, and ions realize thermoextraction and thermointercalation on these active electrodes via thermally driven redox reactions. It should be emphasized that the redox couple utilized in the TEx effect can be regarded as a special type: one component is a solid-state substance intercalation into the active electrode, while the other is the ion extraction in the electrolyte. This mechanism often exhibits pronounced pseudocapacitive behavior, where ion intercalation/extraction triggers significant chemical potential fluctuations at the electrode interface or within interlaminar regions, enabling substantial thermovoltages even under minimal temperature gradients. The thermopower in thermoextraction effect is quantified as


(3) \begin{eqnarray*}
{S}_{{\mathrm{TEx}}} &=& \frac{{\partial E}}{{\partial T}} = - \frac{1}{{nF}}\frac{{\partial {\mathrm{\Delta }}G}}{{\partial T}} = \frac{{{\mathrm{\Delta }}S}}{{nF}}\\
&& = \frac{{\left( {\widetilde {{S}_{\mathrm{B}}} + S_{\mathrm{B}}^*} \right) - \left( {\widetilde {{S}_{\mathrm{A}}} + S_{\mathrm{A}}^*} \right)
- n{\mathop s\limits^{=} }_{\mathrm{e}}}}{{nF}},{\mathrm{\ }}
\end{eqnarray*}

where *E, T, n*, and *F* represent equilibrium potential, temperature, the number of electrons transferred in the electrochemical reaction, and Faraday constant, respectively. $\Delta G$ and $\Delta S$ are the Gibbs free energy and entropy change, $\tilde{S}$ and ${S}^*$ are the partial molar entropies and Eastman entropies of species A and B, ${\mathop s\limits^{=} }_{\mathrm{e}}$ is the total transport entropy of electrons in the external circuit. Crucially, this process is inherently thermally reversible; ions re-intercalate upon the removal or reversal of the temperature gradient, granting TEx systems superior cyclic stability and operational longevity.

Thermoextraction effect—involving selective ion transfer driven by solvation and structural entropy differences—further enhances i-TE performance by coupling ion migration with electrode processes. The interplay of these three i-TE mechanisms enables promising manipulation of ionic transport, entropy flow, and electrode kinetics, offering a versatile strategy to maximize conversion efficiency.

### Category of i-TE materials

An i-TE material is a multi-component system consisting of two primary constituents: (i) an active species that supplies mobile charge carriers, and (ii) a host medium that provides the structural framework and dictates ion transport. The active charge carriers, sourced from a diverse chemical library, may include inorganic salts, acids, bases, ionic liquids (ILs), polyelectrolytes (PEs), or electrochemically active redox couples. Generally, we could use the chemical formula of ‘Matrix-Active Ions’ to describe the i-TE materials, for example, for i-TE aqueous gel: Gelatin (aq.)-KCl-K_4_[Fe(CN)_6_]/K_3_[Fe(CN)_6_] and i-TE aqueous solution: Water-guanidinium chloride (GdmCl)-K_4_[Fe(CN)_6_]/K_3_[Fe(CN)_6_]. Based on the host matrix and the resulting morphological characteristics and properties of the electrolytes, i-TE materials can be primarily classified into four categories: liquid-state, gel-state, PE, and porous solid framework.

#### Liquid state i-TE materials

**Figure 2.**
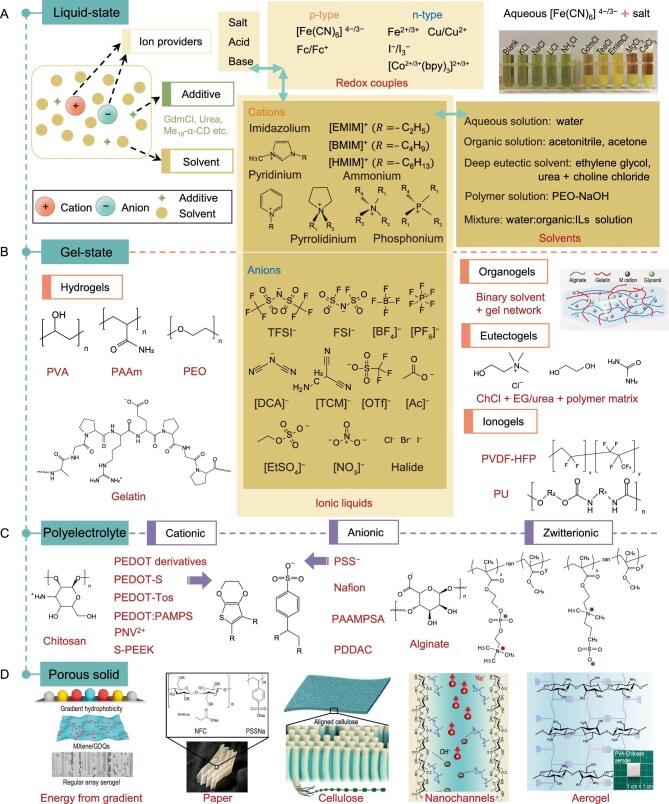
Categories of i-TE materials: liquid-state, gel-state, PE, and porous solid framework. (A) Common liquid-state electrolytes include ion providers, solvents, and additives. Common ions come from salt, acid and base, redox couples, and ILs as well. The appearance and degree of precipitation of aqueous Fe(CN)_6_^4–3–^ solution change when different salts are added. Reproduced with permission from Ref. [38]. Copyright © 2020 The Author(s), exclusive licensee American Association for the Advancement of Science (AAAS). It should be noted that specific ILs can also serve as solvents except for traditional aqueous solution, organic solution, mixture, or novel polymer solutions and deep eutectic with unique characteristics. (B) Gel-state electrolytes can be divided into hydrogels, ionogels, organogels, and eutectogels with the chemical structure of popular ones demonstrated. Reproduced from Ref. [318] under the terms of the Creative Commons Attribution License (CC BY 4.0). Copyright © 2024 The Author(s). Published by Wiley-VCH GmbH. (C) Cationic, anionic, and zwitterionic PEs utilize charged polymer backbones or solid polymer matrices to host mobile ions. (D) Emerging porous solid frameworks provide i-TE electrolytes with nanoconfined channels and advantage diffusion pathways, which can be utilized to capture energy with gradient not limited to heat but also water concentration or moisture. Reproduced with permission from Ref. [123] (Copyright © 2024 Wiley-VCH GmbH); Ref. [126] under the terms of the Creative Commons Attribution 3.0 Unported Licence (CC BY 3.0); Ref. [119] (Copyright © 2019, The Author(s), under exclusive licence to Springer Nature Limited); and Ref. [68] (Copyright © 2023 American Chemical Society (ACS)).

Liquid-state electrolytes have historically served as the cornerstone of the i-TE domain. Over the past decade, they have primarily functioned as model systems to elucidate fundamental mechanisms driven by either the thermodiffusion effect [27,48,49] or the thermogalvanic effect [43,50]. Aqueous electrolytes remain the most extensively studied class of materials for thermogalvanic devices, owing to the high dielectric constant of water, which facilitates ion dissociation, and its low viscosity, which promotes efficient mass transport. As depicted in Fig. 2A, liquid-state i-TE electrolytes are composed of three primary components: ion providers, additives, and solvents. Fundamentally, liquid-state TGCs rely on redox couples, exemplified by the well-established p-type ferro/ferricyanide pair (Water-[Fe(CN)_6_]^4−/3−^), which exhibits a thermopower of approximately +1.4 mV K^−1^ in a 0.4 M aqueous solution [51]. Other common redox couple includes n-type species such as Iron (II/III) (Fe^2+/3+^) [52,53], cobalt (II/III) tris(bipyridyl) [Co(bpy)_3_^2+/3+^] [43,54,55], I^−^/I_3_^−^[52,56–59], Cu/Cu^2+^ [60], with detailed and additional systems discussed in Section ‘Choice of thermogalvanic redox couples’ [61–64]. These conventional aqueous redox systems typically serve as a baseline for further optimization strategies, such as solvation shell restructuring or the introduction of additives [65,66].

In stark contrast to redox-active systems, neat simple salt solutions utilizing the thermodiffusion effect generally exhibit modest thermopower (typically <100 μV K^−1^), often complicated by temporal instability, electrode polarization, and the hydrolysis of multivalent cations [67,68]. In these binary electrolytes, the thermopower is intrinsically governed by the disparity in the Eastman entropies of transfer between cations and anions—a parameter acutely sensitive to hydration thermodynamics, local hydrogen-bond network restructuring, and ion anisotropy. This intricate interplay of ion–solvent and ion–ion interactions frequently leads to non-monotonic concentration dependencies, making the prediction of thermodiffusive behavior challenging. Notably, strongly hydrophilic anions, such as carbonate, were found to induce the significant thermodiffusion required to maximize thermoelectric potential [69].

Beyond aqueous systems, seminal discoveries regarding organic electrolytes [34,70] and aqueous PE solutions [36] have demonstrated that thermodiffusion can yield substantial thermoelectric potentials, highlighting the vast potential of complex liquid-state systems. To explain the divergence from the moderate thermopower of simple salts, theoretical work by Würger elucidates that in complex ionic conductors containing mobile molecular components (for example, water or PEG), a diffusiophoretic transport mechanism driven by thermally induced concentration gradients of these neutral species can significantly amplify the thermoelectric response [40]. This mechanism, operating alongside a ratchet effect that rectifies activated ion hopping under a temperature gradient, provides a comprehensive theoretical framework for the giant thermopower observed in ILs and hydrated polymer electrolytes, thereby delineating a pathway for designing high-performance thermoelectric materials beyond simple electrolytic solutions.

From another perspective, the primary advantages of aqueous electrolytes lie in their high ionic conductivity, cost-effectiveness, and environmental friendliness. However, their application is significantly constrained by the intrinsic physical properties of water, specifically a narrow operating temperature range (<100°C) and long-term stability challenges associated with evaporation, leakage, a limited electrochemical stability window, and potential electrode corrosion. To address these limitations, solvent mixtures have been explored, offering a pathway to tunable thermoelectric properties via the modulation of solvent polarity, viscosity, and solvation structures [71]. Nevertheless, their practical application is similarly hindered by stability challenges related to leakage, necessitating rigorous encapsulation, and they often exhibit lower power factors compared to pure systems. Although organic solvents can offer high thermopower, their practical utility is often compromised by their volatility such as acetone and dimethyl sulfoxide, safety concerns, and relatively low ionic conductivity of the resulting electrolytes [70]. ILs present another alternative, characterized by low thermal conductivity within the matrix and superior long-term stability with electrodes [72]. Furthermore, deep eutectic solvents (DESs) have emerged as promising candidates to bridge the operational temperature gap between aqueous (<100°C) and high-temperature molten salt systems (400–600°C). Formed by the complexation of a hydrogen bond donor (for example, ethylene glycol (EG), urea) and a hydrogen bond acceptor (for example, choline chloride), DESs share the low volatility of ILs but are generally more cost-effective, easier to synthesize, less toxic, and operational in the mid-temperature range (135–165°C) [73].

#### Gel state i-TE materials

The immobilization of ions within polymer networks (hydrogels, ionogels, or elastomers) imparts dimensional stability while enhancing the thermopower through polymer–ion interactions and nanoconfinement. As shown in Fig. 2B, synthetic hydrogels serve as versatile platforms: polyvinyl alcohol (PVA) matrices leverage high hydroxyl density for redox hosting, where salting-out strategies and coordination chemistry optimization yield giant thermopowers [74]. To overcome trade-offs between mechanics and transport, hybrid approaches employ secondary components such as starch for thermal stability or conductive nanofillers (for example, MXene) for multi-modal sensing [75,76]. Similarly, polyacrylamide (PAAm) is preferred for tough, stretchable devices, utilizing ‘water-in-salt’ or binary solvent strategies to suppress ice crystallization and double-network architectures for self-healing [77,78].

Biological matrices offer unique entropy-driven advantages. Gelatin exploits the Hofmeister effect to modulate hydrophobic–hydrophilic interactions, significantly enhancing thermopower, though its thermal stability remains limited [78,79]. Lignin-derived hydrogels have also achieved high figures of merit via alkaline electrolyte infiltration [80]. Polysaccharides, particularly bacterial cellulose (BC), utilize hierarchical nanofiber networks to create tortuous ion paths. Strategies include 2,2,6,6-tetramethylpiperidine-1-oxyl radical (TEMPO)-oxidation for IL encapsulation and mesoscopic confinement to enhance the TD effect [81,82]. Furthermore, methylcellulose (MC) enables entropy-driven thermopower enhancement at low concentrations [83]. While generally less elastic than synthetics, polysaccharide mechanics can be reinforced via interpenetrating networks (for example, with cellulose nanofibrils) or double hydrogen-bonding systems [84–86].

Ionogels immobilize ILs within solid matrices, effectively mitigating leakage issues while modulating thermoelectric performance through host-guest interactions. Typical cationic and anionic structures are illustrated in Fig. 2A and B. Among various matrices, fluoropolymers such as poly(vinylidene fluoride-co-hexafluoropropylene) (PVDF-HFP) serve as performance benchmarks; their strong ion–dipole interactions promote ion pair dissociation and selectively hinder the thermodiffusion of specific ions, yielding high thermopower (for example, 26.1 mV K^−1^) [87]. Representative fluoropolymer-based systems include PVDF-HFP-EMIM:DCA [87], PVDF-HFP (ethanol treated)-EMIM:DCA [88], PVDF-HFP-EMIM:DCA-Na:DCA [87], PVDF-HFP-EMIM:DCA-Na:TFSI [89], PVDF-HFP-Ag:OTf-BMIM:BF_4_ [90] and ternary blends such as PVDF-HFP/PEG-EMIM:TFSI [58]. Beyond fluoropolymers, alternative matrices have also attracted significant attention, including PVA-based (for example, PVA-EMIM:DCA [91], PVA-EMIM:Cl [92]), polyurethane (PU) based (for example, waterborne polyurethane (WPU)-EMIM:DCA [93], PU-EMIM:DCA [94]), PEO-based (for example, PEO-EMIM:Ac [95], PEO/P123-EMIM:OAC [95]), biopolymer-based (for example, BC-EMIM:DCA [96], Cellulose-AMIM:Cl-ZnCl_2_ [97], Gelatin-EMIM:OAC-CH_3_COONa [98]) and composites such as poly(methyl methacrylate-*co*-methyl acrylate) [P(MMA-*co*-MA)]/MXene-EMIM:TFSI [99]. Detailed illustration and thermoelectric properties can refer to the summarized table in Section ‘Choice of thermodiffusion ions’.

Hierarchically porous networks further decouple mechanical support from ion transport [88], while synergistic doping allows bidirectional thermopower tuning [90]. Biopolymer ionogels (for example, gelatin and cellulose) alternatively leverage supramolecular assembly for ion-selective channels. To enhance functionality, nanocomposite strategies incorporate fillers such as SiO_2_ or ZIF-8, which act as Lewis acids or sieving channels to amplify free carrier concentration and tune transportation behaviors [100–102]. For wearable applications, PU-based ionogels achieve high stretchability (300%), with studies unexpectedly identifying atmospheric moisture as a conductivity enhancer [94].

To broaden the operating temperature envelopes, binary solvent-based organogels, such as H_2_O/EG-N, N-dimethylamino ethylacrylate (DMAEA-Q)/acrylamide (AM)-Fe^2+/3+^, H_2_O/glycerol (Gly)-PVA/2-(6-isocyanato-hexylamino)–6-methyl-4[1H]-pyrimidone (UPy)-ZnSO_4_ and H_2_O/propylene glycol (PG)-BC-FeCN^4−/3−^, have been developed to suppress ice crystallization, thereby enabling flexibility and energy harvesting [103–105]. Notably, the former two systems leverage synergistic chaotropic effects to optimize thermogalvanic processes even under deep-freezing conditions (down to −50°C) [103]. In parallel, eutectogels based on DESs, including choline chloride (ChCl)/EG-WPU, ChCl/EG-cellulose nanofibril (CNF)/PVA-Sodium Tetraborate Decahydrate (Borax), BC/polyacrylic acid (PAA)-GdmCl-Fe(CN)_6_^4−/3−^ (or Fe^2+/3+^), effectively mitigate aqueous volatility via robust hydrogen-bonded networks [73,106,107]. This strategy holds the potential to extend the operating temperature range to regions previously inaccessible to conventional hydrogels.

#### PE i-TE materials

Moving beyond liquid and gel-based systems, PE materials could be considered as another family of i-TE materials without arbitrary introducing the salt or IL. These materials utilize charged polymer backbones or solid polymer matrices to host mobile ions, eliminating leakage risks and enabling the fabrication of flexible, printable, and compact energy harvesters. Unlike traditional electronic conducting polymers where the thermoelectric effect is driven by charge carrier diffusion, PE systems (Fig. 2C) primarily harness the thermodiffusion of ions or redox-entropy changes associated with polymer-pendant groups.

While conducting polymers such as PEDOT:PSS are typically valued for their electronic conductivity, recent investigations have decoupled and highlighted their i-TE contributions. The complex interplay between electronic and ionic transport in these systems allows for the tuning of the net thermopower. For instance, post-deposition processing has been shown to modulate the film microstructure, thereby influencing the relative contributions of the electronic and ionic thermopowers [108]. Shu *et al*. demonstrated that the ionic thermopower of PEDOT:PSS PE films can be tuned from −9.63 to +3.07 mV K^−1^ by manipulating the cation softness parameter of incorporated inorganic chlorides, which governs the binding strength between cations and the PSS chains to determine the dominant mobile charge carriers [109]. True solid-state PEs eliminate liquid components entirely, relying on ion hopping or transport through polymer chains (for example, Nafion, PEDOT:PSS). While they traditionally offer lower ionic conductivity than gels, they excel in mechanical strength and processability. By introducing mixed ionic and electronic components, Cho *et al*. developed a transparent, self-healable mixed ionic-electronic conductor (MIEC) using a PVA/PEDOT/poly(2-acrylamido-2-methyl-1-propanesulfonic acid) (PAMPS) hydrogel. In this polymer composite, PAMPS provides an ion channel for anion carriers, which are the major carriers in the TE harvester, while PEDOT serves as an electronic channel to realize a mixed ionic-electronic TE, which achieves a high n-type ionic thermopower through the selective transport of biodegradable bisulfate anions [110].

Furthermore, the humidity-dependent behavior of these PE materials reveals that ionic transport often dominates at high moisture levels, where the hydration of the sulfonate groups in PSS facilitates ion mobility [111,112]. Hygroscopic PE complexes, such as poly(styrene sulfonic acid) (PSSA)-PEDOT:PSS-FeCN^4−/3−^, leverage moisture absorption to promote ion dissociation [113]. The huge mobility difference between the mobile cations Na^+^ and the anchored polyanions results in giant ionic thermopower, with values reaching −33 mV K^−1^ [114]. This dual-nature mechanism has been explored in acid-doped systems as well, such as hydrochloric acid-doped polyaniline (PANI), where the thermoelectric properties are governed by the protonation level of the polymer backbone [115]. In addition, Zhang *et al*. [116] demonstrated a colossal thermal-to-electrical conversion factor in a polyaniline:poly(styrene sulfonate) (PANI:PSS) solid-state PE by exploiting the thermodiffusion of water molecules to modulate the electrochemical corrosion potential of carbon steel electrodes, distinct from the traditional TD effect. The challenge in this sub-category remains balancing the high electronic conductivity required for charge collection with the high ionic thermopower required for voltage generation, a trade-off that is often managed through precise doping and humidity control.

Alternative PE architectures have been developed to introduce multifunctionality and novel transport mechanisms. Ternary hybrid systems, such as PANI:poly(acrylamide-co-2-acrylamido-2-methylpropane sulfonic acid) (PAAMPSA): phytic acid (PA) [117], harness dynamic electrostatic and hydrogen-bonding networks to facilitate proton thermodiffusion, simultaneously achieving intrinsic self-healing and high stretchability. To address the scarcity of n-type counterparts to PSS-based materials, cationic polymers poly(diallyldimethylammonium) chloride (PDADMAC) or poly(2-(dimethylamino)ethyl methacrylate) methyl chloride quaternary salt (MADQUAT) [116] utilizes anion exchange to modulate hydration energy and mobility, yielding air-stable, solution-processable films. Further, dynamic redox-responsive systems such as poly(N-isopropylacrylamide-co-vinylferrocene) (PNV) move beyond static PEs [118]. By integrating ferrocene redox centers into a thermo-responsive backbone, PNV couples the electrochemical potential with coil-to-globule phase transitions, effectively exploiting large entropic changes near the lower critical solution temperature to maximize thermopower.

#### Porous solid framework i-TE materials

Recent advancements have highlighted the use of rigid, nanoporous scaffolds for electrolyte confinement. Crystalline metal-organic frameworks (MOFs), alongside wood-derived structures, modified paper, and aerogels, offer highly ordered nanochannels for ion transport [68,119–121].

As illustrated in Fig. 2D, by retaining the intrinsic vertically aligned nanofibers and mesoporous framework of natural wood, the engineered cellulosic membrane presents a scalable strategy for fabricating charged nanochannels [119]. These sub-nanometer confined spaces facilitate surface-charge-governed ion diffusion, significantly enhancing thermal voltage generation via nanoconfinement effects [122]. A hierarchical porous framework of conductive MOF nanolayers on cellulose nanofibers leverages charged nanochannels and exceptional photothermal properties to synergistically couple ionic thermophoresis with evaporation-induced streaming potentials, yielding remarkable sustained open-circuit voltage and power density under one sun [120]. Similarly, a lotus-inspired hierarchical porous solid framework, comprising a chitosan/carboxymethyl cellulose hydrogel matrix functionalized with graphene quantum dots/MXene nanocomposites and a gradient hydrophobic coating, synergistically couples evaporation-induced streaming potentials with ionic thermodiffusion across charged nanochannels to achieve a single-unit power density of 45.6 μWcm^−2^ and a scalable system alongside efficient freshwater harvesting [123].

Lignin-derived ionic conducting membrane, possessing hierarchical aligned channels and nanosized confinements within these channels, exerted excellent thermal stability and superior swelling ratio, and remarkable i-TE performance by selective ion diffusion [124]. An all-wood ionic gel was developed by employing a lignocellulose channel reconstruction strategy, utilizing urea/alkali-mediated molecular integration of sodium lignosulfonate within a regenerated cellulose framework to create spatially confined anionic sites, which effectively decouples ionic thermodiffusion for selective cation transport [125].

In addition, employing paper as the transportation media for ions offers a route for the mass production of large-area i-TE devices. Self-powered, foldable thermoelectric paper chips were developed by integrating p-type EMIM:Ac and n-type EMIM:TFSI ILs within a porous paper matrix, leveraging differential ion adsorption/desorption on gold electrodes to achieve a significant thermoelectric response for early fire alarm applications [121]. As another example, utilizing nanofibrillated cellulose to mechanically reinforce a polystyrene sulfonate sodium matrix, Jiao *et al*. [126] developed a robust i-TE paper with an elevated thermopower of 8.4 mV K^−1^, where the retention of high-boiling-point solvents within interstitial nanovoids preserves efficient ionic percolation pathways. Regarding aerogel-based architectures, a PVA-chitosan solid framework infiltrated by CuCl_2_-doped polycationic poly(diallyldimethylammonium chloride) (PDDA) was developed to exhibit high ionic conductivity and thermopower [68]. The synergistic cation-anchoring mechanism, driven by Cu^2+^-chitosan coordination and PDDA-induced Coulombic repulsion, facilitates the selective transport of [CuCl_4_]^2−^ anions, offering a significant strategy for designing i-TE materials through complex ion–polymer interactions.

### Thermoelectric properties

The fundamental performance of any thermoelectric material, whether based on electronic or ionic charge carriers, usually requires good electrical conductivity and poor thermal conductivity, in addition to high thermopower. However, the ion could not pass through the electrode, leaving the direct current (DC) conductivity without a proper definition for most ionic materials. In other words, the DC electrical conductivity is not yet a good scale for the i-TE materials. In this section, we will discuss the measurement and some insights into thermoelectric parameters.

#### Thermopower, measurement, and intrinsic/non-intrinsic thermopower

Thermopower is an intrinsic physical parameter of uniform thermoelectric materials. For the i-TE materials, thermopower includes contributions from both the thermodiffusion and thermogalvanic effect of ions. Experimentally, there are two topological configurations (vertical or planar) used for thermopower measurements of i-TE materials, which impose distinct boundary conditions on the measurement. As illustrated in the standard vertical measurement apparatuses (Fig. 1C), the material is confined within a non-conductive spacer between parallel electrodes, a geometry that facilitates the necessary encapsulation against solvent evaporation for liquid or gel-based electrolytes. This spacer can also protect the tested material from deformation in case of any thermovoltage disturbance. The temperature difference across the tested material is generally generated from two Peltier chips or other thermostatic plates with precise temperature control. The thermopower *S*_i_  $= - \frac{{{V}_{\mathrm{H}} - {V}_{\mathrm{C}}}}{{{T}_{\mathrm{H}} - {T}_{\mathrm{C}}}}$ can be achieved by linear fitting of a series of temperature–thermovoltage pairs to remove the voltage drift at $\Delta T$ = 0. Usually, 5–10 points of $\Delta T$ were used to minimize the error bar. A small $\Delta T$ usually corresponds to a large error bar.

While planar architectures are ubiquitous in thin-film characterization, they introduce a significant metrological discrepancy stemming from the spatial mismatch between the potentiometric probe spacing (${d}_{\mathrm{1}}$) and the thermal probe spacing (${d}_{\mathrm{2}}$). Although geometric correction factors are routinely applied to derive the thermopower via* S*_i_ = $- \frac{{\Delta V}}{{\Delta T}} \times \frac{{{d}_{\mathrm{2}}}}{{{d}_{\mathrm{1}}}}$, the validity of this approach is strictly contingent upon the linearity of the thermal gradient across both the substrate and the i-TE material. Consequently, any deviation from this idealized profile can compromise the correction, introducing significant artifacts into the reported thermopower.

Beyond geometric constraints, the validity of i-TE characterization is frequently compromised by environmental instabilities and interfacial electrochemical artifacts. For instance, in open-boundary planar setups, the lack of rigorous humidity control can lead to solvent evaporation at the heated side, inducing a local concentration spike (${\mathrm{\Delta }}C$) that generates a spurious Nernst potential ${V}_{{\mathrm{total}}} = S{\mathrm{\Delta }}T + \frac{{RT}}{{nF}}\ln ( {\frac{{{C}_{{\mathrm{hot}}}}}{{{C}_{{\mathrm{cold}}}}}} )$, that should not be considered as an intrinsic contribution for thermopower, as it is unrelated to the TD effect. This hygroscopic instability artificially inflates the observed voltage, conflating thermal diffusion with drying-induced concentration gradients. Furthermore, the thermopower can also be affected by electrode polarization, contact potential, electrode corrosion, Donnan exclusion effect, and electrostatic adsorption at the electrolyte–electrode interfaces [127–129].

#### Electrical conductivity, measurement, and frequency dependence

The electrical conductivity is calculated via $\sigma = \frac{{\mathrm{1}}}{R}\frac{l}{A}$, where *R* is the resistance of a sample, *l* and *A* are the length (or thickness) and the sample’s cross-sectional area, respectively. Resistance measurements typically employ DC or alternating current (AC) methods using four-point (for low resistance) or two-point (for intermediate and high impedance) configurations. In liquid-phase electrochemical impedance spectroscopy (EIS), a three-electrode cell with a potentiostat is typically used to control the working electrode potential relative to a reference.

Device-level metrics, such as effective conductivity (${\sigma }_{{\mathrm{eff}}}$) derived from steady-state current–voltage (*I–V*) sweeps, offer practical insights into power output but fail to isolate material-intrinsic properties from interfacial losses (charge-transfer resistance ${R}_{{\mathrm{ct}}}$ and mass transport resistance ${R}_{{\mathrm{mt}}}$). This limitation is critical in systems with blocking electrodes, where DC-induced polarization and screening fields compromise data fidelity. To rigorously extract intrinsic conductivity ${\sigma }_{\mathrm{i}}$, EIS is preferred. By probing the system at high frequencies (∼100 kHz), EIS confines ion motion to local oscillations, thereby bypassing the concentration polarization inherent to DC techniques. The bulk resistance (*R*_b_) is theoretically identified at the high-frequency intercept of the Nyquist plot. However, the implicit nature of frequency in Nyquist diagrams complicates the demarcation between bulk and interfacial responses. This challenge is particularly acute in soft matter electrolytes, where dynamic changes in hydration and temperature can morph the ideal vertical capacitive tail into a slanted spur dominated by Warburg diffusion or non-ideal constant phase element effects, risking erroneous extrapolation of ${\sigma }_{\mathrm{i}}$. Therefore, reporting ionic conductivity requires validating the existence of a plateau in the Bode plot for every temperature point measured. Additionally, the rise of MIEC materials (for example, doped PEDOT:PSS, carbon nanotube (CNT)-ionogels) necessitates advanced characterization protocols to decouple ionic (${\sigma }_{\mathrm{i}}$) and electronic (${\sigma }_{\mathrm{e}}$) conductivities. While standard AC impedance spectroscopy effectively captures the aggregate response at high frequencies, it often fails to deconvolute the individual components, particularly when carrier relaxation timescales overlap or when the electronic pathway effectively shorts the ionic capacitive response. To address this, the Hebb–Wagner polarization method is widely employed as the definitive DC technique. Through an asymmetric cell design comprising an ion-blocking electrode and a reversible counter electrode, this method allows for the precise isolation of the electronic contribution by measuring the steady-state current.

To advance the design of i-TE materials, intensified research efforts are warranted to elucidate the mechanistic interplay between activation energy (${E}_{\mathrm{a}}$), ionic conductivity and thermopower. By analyzing temperature-dependent conductivity via Arrhenius or Vogel–Fulcher–Tammann formalisms, one can extract ${E}_{\mathrm{a}}$ as a thermodynamic fingerprint of the ion transport mechanism. In ionic conductors, empirical evidence and theoretical models suggest that heat of transport is often comparable in magnitude to the activation energy [130]. Complementing this thermodynamic perspective, equivalent circuit modeling remains indispensable for quantifying kinetic parameters. TGCs are frequently approximated by the Randles model [69], allowing for precise extraction of internal resistance components. Furthermore, the interfacial charge transfer kinetics are governed by the Butler–Volmer equation [131]. Recent advances have extended EIS models to encompass both isothermal and non-isothermal regimes of TGCs [132]. The formulation of high-fidelity equivalent circuits that explicitly incorporate thermal gradients is instrumental in unraveling reaction mechanisms and rationalizing component optimization from an electrochemical standpoint.

Noted that there is still a lack of a standard electric conductivity for the i-TE materials, which could accurately connect the materials-level properties with device-level power output performance. However, the electrical conductivity measured from the EIS method, or steady-state I–V sweeps, could still be effective to qualitatively guide the material optimization.

#### Thermal conductivity, measurement, and conventional impact

Suppressing thermal conductivity is critical to sustaining the temperature gradient that drives ionic transport. In i-TE systems, ILs and gels lack long-range translational order, whose structural architecture of soft matter provides a natural advantage in phonon engineering. Their thermal transport is dominated by short-range intermolecular energy transfer, vibrational modes of disordered networks, and in the case of liquids, molecular collisions. The thermal conductivity of ILs is generally low, typically between 0.1 and 0.2 W m^−1^ K^−1^, which is significantly lower than water (0.6 W m^−1^ K^−1^). However, gel-state materials typically exhibit thermal conductivities ranging from ∼0.15 to 0.5 W m^−1^ K^−1^, values that are intermediate between those of their constituent liquid and polymer phases.

In liquid systems, natural convection creates an additional and highly efficient pathway for heat transfer, which is counterproductive to maintaining a temperature gradient even though help facilitate mass transfer. Further optimizing thermal conductivity is challenging because common strategies to reduce $\kappa $ (for example, increasing microporosity, and incorporating inorganic nanoparticles or crystalline domains) often offer limited efficacy while simultaneously compromising ionic conductivity by obstructing ion transport pathways. While high-performance i-TE materials are best realized by decoupling ionic conductivity from thermal transport, accurately quantifying the latter remains non-trivial. Given the diversity of ionic conductors, thermal characterization demands methodologically specific protocols rather than a ‘one-size-fits-all’ measurement protocol.

Thermal conductivity characterization of i-TE materials is dictated by phase state and geometry, yet standard methods face unique constraints in soft matter. The transient hot-wire method, typically used for fluids, suffers from parasitic electrical conduction in electrolytes. Accurate implementation therefore requires electrically insulating sensors (for example, via parylene) to prevent electrolysis while minimizing thermal resistance. Moving to frequency-domain techniques, the 3ω method is advantageous for rigid solids and thin films. The thermal conductivity of measured material is extracted from the frequency dependence of the in-phase temperature oscillation amplitude. The thermal conductivity of hydrogel or ionogels are significantly affected by the water content, which is usually dismissed in literature. Taking 3ω method as an example, simulations indicate that for short measurement times (<2 s) with appropriate encapsulation, the error due to evaporation can be kept below 1%, whereas open systems would unacceptably alter the value of the thermal conductivity of the material due to water loss [133]. The implementation of a bidirectional 3ω technique was utilized to measure the thermal conductivity of PAAm and PAMPS hydrogels with particular attention to their moisture content [133]. For bulk solids, laser flash analysis is standard but struggles with i-TE materials due to optical transparency and evaporation, necessitating opaque coatings and complex multilayer analysis. Conversely, steady-state methods (for example, guarded hot plate) are theoretically direct but practically limited by long equilibration times, which risk dehydrating hydrogel samples.

### I-TE devices

#### Device morphology: single-cell and dual-cell

**Figure 3.**
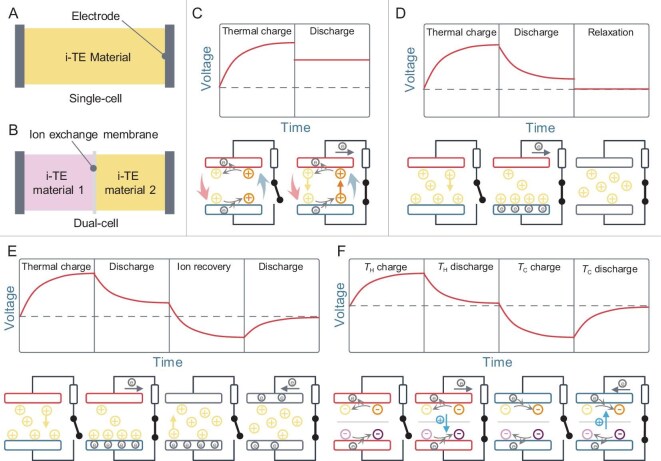
Two types of i-TE cells and i-TE working modes. (A) Single-cell. (B) Dual-cell. (C) Continuous i-TE generator mode. (D) Intermittent i-TE generator mode. (E) I-TE capacitor mode. (F) Self-regenerating i-TE cycle mode. The electrodes are shaded according to their thermal state: the hot electrode (relatively high temperature), the cold electrode (relatively low temperature), and the electrode at ambient temperature.

Typical i-TE devices are usually composed of an i-TE cell or multiple cells integrated in series and parallel connection. An i-TE cell primarily comprises electrodes, i-TE materials, and packaging materials. Based on their structural characteristics, i-TE cells can be classified into two types: single-cell and dual-cell, as shown in Fig. 3A and B, respectively.

The i-TE single-cell is composed of a specific i-TE material in which ions are transported under a temperature gradient, or an ionic redox reaction occurs, thereby converting thermal energy into electrical energy. Such devices are typically used to harvest the thermal energy characterized by a temperature difference on a spatial scale, and the relative positions of electrodes and electrolytes vary according to material properties and application scenarios. Typical structures include sandwich structures and planar structures. The i-TE dual-cell consists of two different i-TE materials, which usually involve two types of redox couples. The two half-cells are generally separated by an ion-exchange membrane, enabling the transport of active ions from the two redox couples in their respective electrolytes and their redox reactions at the electrodes. Unreacted ions with opposite charges can transport between the two electrolytes through corresponding types of ion-exchange membranes to balance charge and ion concentrations on both sides, thereby enabling the device to operate continuously. Unlike single-cell, dual-cell can generate electrical output without a spatial temperature difference between the electrodes, enabling heat recovery and energy storage based on a temporal temperature difference. Table 1 delineates the key differences between single-cell and dual-cell batteries, focusing on four critical aspects: structural design, performance characteristics, operational modes, and primary applications.

**Table 1.** Comparison between i-TE single-cell and dual-cell.

	I-TE single-cell	I-TE dual-cell
Structural characteristics	Single i-TE material Single sandwich/planar cell	Two i-TE materials Two half-cells separated by IEM^a^
Key performance metrics	Thermopower Thermal conductivity Long-term output energy density	Thermopower Thermal capacity Cycle capacity
Working modes	I-TE generator mode I-TE capacitor mode	I-TE cycle mode
Working conditions and application	Spatial temperature difference Instant thermoelectric conversion	Temporal temperature difference Thermoelectric conversion and storage

a IEM, ion exchange membrane.

#### I-TE single-cell: working modes and major performance metrics

Single-cell i-TE devices exhibit diverse operational behaviors depending on their electrolyte (liquid vs. quasi-solid) and the dominant mechanism (thermogalvanic vs. thermodiffusion). The main working modes include continuous i-TE generator mode, intermittent i-TE generator mode, and i-TE supercapacitor mode. The continuous i-TE generator mode is characteristic of liquid-based cells operating primarily via the thermogalvanic effect (Fig. 3C). After an initial thermal charging period, the device enters a discharge state. Convective flow in the liquid electrolyte facilitates continuous mass transport of redox species, enabling a prolonged, relatively constant voltage output under a constant load resistance. The output power in this steady state, however, is often lower than the maximum instantaneous power derived from *I–V* sweep. The intermittent i-TE generator mode is adopted by quasi-solid-state gels or systems where ion transport is slow, often involving a significant thermodiffusion contribution (Fig. 3D). The output voltage decays continuously during discharge. A single cycle consists of three stages: (i) thermal charging under a temperature gradient to build up thermovoltage; (ii) discharge to an external load; and (iii) removal of the temperature gradient and short-circuiting of the electrodes to reset ion distribution and electrode charge. Moreover, the i-TE supercapacitor mode leverages the device’s intrinsic capacitance for thermal energy storage and subsequent electricity generation without concurrent thermal input (Fig. 3E). A full cycle encompasses four stages: (i) and (ii) are identical to the intermittent generator mode; (iii) the temperature gradient is removed while the circuit remains open, allowing the internal ion concentration gradient to dissipate, which causes a decay and potential reversal of the open-circuit voltage; and (iv) the electrostatically stored energy is discharged through a load.

Although the working mechanisms are different, their core performance indicators for single-cell i-TE devices must account for their transient and cyclic nature, including maximum instantaneous output power density, long-term output energy density, and thermoelectric conversion efficiency. The maximum instantaneous output power density (*P*_max_) is typically calculated from the *IV* curve of the cell, obtained through variable load scanning. While useful for rapid screening, it is an inadequate standalone metric, as i-TE cells typically cannot sustain continuous power output at the maximum instantaneous power density without decay. Therefore, this metric is insufficient for characterizing continuous operation or calculating thermoelectric conversion efficiency. A comprehensive evaluation of practical devices requires that it be complemented by the energy density per cycle. Considering the unique slow thermal charging process and continuously decaying output characteristics of i-TE devices, their working modes are usually periodic. Using the output energy per cycle (*E*_cycle_) as the key output performance indicator to calculate thermoelectric conversion efficiency is more convincing. This is a more relevant metric, defined as the total electrical energy delivered to the load during one complete discharge phase (Stage II in intermittent mode, or Stage IV in capacitor mode). It reflects the practical energy yield of a single operational cycle. Moreover, when the discharge time is much longer than the charging time, the thermoelectric conversion efficiency (*η*), which is the most direct performance metric used in all energy conversion technologies, should be evaluated over a complete cycle. It is the ratio of the total electrical energy output (*E*_cycle_) to the total thermal energy input required to establish and maintain the temperature gradient during the thermal charging and discharge phases. For the intermittent mode, thermal input occurs only in Stages I and II. The commonly used electronic figure of merit (*ZT*) is inappropriate due to the fundamental differences in charge carrier dynamics and the non-steady-state power output of i-TE systems.

#### I-TE dual-cell: working modes and major performance metrics

The i-TE dual-cell devices based on the thermogalvanic effect, offer a unique working mode tailored for energy storage over time, utilizing temperature variations (Fig. 3F). This is the hallmark mode for dual-cells, enabling operation under a temporal temperature difference. The cycle leverages the distinct temperature coefficients (*α* = d*E*/d*T*) of the two different redox couples. Noted that temperature coefficient has the same thermodynamic insight of thermopower but opposite due to a difference in definition.

At high temperature condition (${T}_{\mathrm{H}}$), a potential difference is established between the two half-cells, allowing for electrical energy output to a load. During discharge, the concentration ratios of the redox species in the two half-cells change with the load voltage decreasing to zero. When the device is cooled to a lower temperature (${T}_{\mathrm{C}}$), due to the difference in temperature coefficients of the two redox couples, the open-circuit voltage of the device decreases or even becomes negative. Subsequently, when connecting a load to output electrical energy, the direction of the output current is opposite to that at high temperature, and the relative concentration ratio of redox couple ions in the two half-cells is recovered. Through high-temperature thermal charging and discharging, as well as low-temperature thermal charging and discharging, thermal energy is converted into chemical energy for energy storage in the second and fourth stages, thereby enabling external energy output, ion regeneration, and balance under temporal temperature-difference conditions. The performance evaluation for dual-cells in the cycle mode focuses on the energy converted over a full temperature swing, including cycle energy density, thermoelectric conversion efficiency. The cycle energy density indicates the sum of the electrical energy output during both the high-temperature and low-temperature discharge stages. The efficiency for a complete cycle is defined as the ratio of the total electrical work output (sum of work from ${T}_{\mathrm{H}}$ and ${T}_{\mathrm{C}}$ discharges) to the total thermal energy input. This input includes the heat absorbed to raise the device temperature from ${T}_{\mathrm{C}}$ and ${T}_{\mathrm{H}}$ and the heat required to maintain the system at ${T}_{\mathrm{H}}$ during the discharge period.

## CHALLENGES IN CHEMISTRY OF I-TE

### Choice of thermodiffusion ions

The active ionic species fundamentally govern the functionality of i-TE materials, as upon application of a temperature gradient, cations and anions within the i-TE materials undergo selective migration [25]. Usually, ions with small radii and low solvation energies (for example, Na⁺, Cl⁻) typically exhibit rapid diffusion, whereas larger ions with robust solvation shells or intricate structural motifs (for example, EMIM⁺, TFSI⁻) migrate more sluggishly. This differential mobility establishes an ionic concentration gradient across the system. Consequently, an asymmetric ion distribution develops between the hot and cold terminals, leading to the formation of an electric double layer (EDL) at the electrode interfaces. The EDL comprises a compact inner Helmholtz layer and an extended diffuse layer, ultimately generating a measurable thermovoltage between the electrodes [14,134]. Thus, strategic selection of thermodiffusion ions and precise modulation of cation/anion mobility disparities represent central approaches for enhancing i-TE device performance. These principles also establish the chemical foundation for designing both simple-ion and complex-ion systems, as summarized in Table 2 and elaborated in subsequent subsections.

**Table 2.** Summary of the compositions and key properties of selected i-TE materials.

Type	Ion donors	Matrixes	*S* [mV K^−1^]	*σ* [mS cm^−1^]	*κ* [W m^−1^ K^−1^]	Stability	Ref.
Simple ions	NaOH	PEO^(^^1)^	11.1	0.08	0.22	Non-volatile up to 120°C; ∼73% self-discharge	[36]
	NaOH	Cellulose/PEO	24.0	20	0.48	Thermally stable (<315°C); rapid thermal charging (∼70 s)	[119]
	NaOH	PVA^(^^2)^	−37.6	0.07	0.42	Stable voltage for 6+ hours and performance for 15 days; stable coordination interaction	[136]
	NaOH	PQ-10^(^^3)^	24.17	10	–	Stable sensor sensitivity over 57 days	[135]
	NaOH	PAMPS^(^^4)^/PSBAA^(^^5)^	31.7 ± 0.39	60 ± 1.12	0.5 ± 0.006	Stable quasi-solid state; pH-stability of i-TE at pH 1	[324]
	NaOH	PVA/TOBC^(^^6)^	−20.65	0.62	–	Thermally stable as a quasi-solid (<136.7°C)	[325]
	NaI	PVA	42.8	51.5	0.54	100+ charge–discharge cycles with high stability; repeated bending/distortion without delamination	[168]
	NaCl	PAAM^(7)^/CMC^(^^8)^-Na	17.1	26.8	–	Cycling stability as a supercapacitor (86.1% capacitance retention over 7500 cycles); fully reversible i-TE performance via rehydration	[326]
	NaCl	Coordinated	−27.2	204.2	–	High structural stability via irreversible Ca^2+^ coordination	[82]
	NaCl	PAAM/PDA^(^^9)^/CNT-COOH^(^^10)^/PANI^(11)^	18.6	175.3	0.68	Wearable use (>70% strain); elasticity and structural integrity over 10–60°C	[327]
	NaBF_4_	PVA/SA^(12)^/PEG^(13)^	66.7	31.4	–	Tensile stress and strain up to 69 kPa and 114%; 15% strain wearable use	[328]
	LiCl	Polyacrylamide/poly(sodium alginate)	10.5	10	–	Performance deteriorates >50°C; short-circuit current stability of i-TE enhanced TENG (>5000 cycles); 1655% fracture strain	[329]
	CsI	PVA	52.9	51.5	0.54	Reproducible performance across laboratories	[168]
	KOH	Lignin	30.4	5.9	0.36	Being resistant to simple cutting, crushing, and stretching stress tests	[330]
	CuCl_2_	PVA/Chitosan	28.4	40.5	0.49	Robust under fluctuating thermal waveforms; favorable biodegradability and environmental compatibility	[312]
	CuCl_2_	PEDOT:PSS^(^^14)^	−18.2	52.6	0.34	Stable > 30 days (80% RH); 96.5% *V*_oc_ and 95% *C*_s_ retention after 10-day ambient storage	[207]
	ZnSO_4_	PVA/UPy^(^^15)^	−3.69	1.5	–	*T*_m_ ≈ 225°C; stable >120 min (encapsulated); performance maintained under repeated 10% strain, 90° bending, compressing, and cutting	[331]
	H_3_PO_4_	PVA	1.6	2.9	–	*S* maintained across 30–70% RH	[332]
	H_3_PO_4_	PANI/PAAMPSA^(^^16)^	8.1	237.0	0.48	Sustainable i-TE performance under severe deformation (50% strain) and multiple self-healing processes (30 cycles)	[117]
	HCl	PAMPS/PSBAA	−32.6 ± 0.79	60 ± 1.12	0.5 ± 0.006	Stable quasi-solid state; pH-stability of i-TE at pH 14	[324]
	HCl	PVA	38.2	18.87	0.458	Higher thermal stability (hydrogel stretched for 48 h) in the range of 25–250°C; i-TE performance retained over 4 days	[230]
Complex ions	[EMIM:DCA]	PVDF-HFP	26.1	6.7	0.18	Thermally stable (<85°C); flexible, leak-proof quasi-solid structure	[87]
	[EMIM:DCA]	PVA	4.67	∼11.7	0.28	Thermally stable (−20–75°C); 2.03 MPa strength with high toughness	[91]
	[EMIM:DCA]	WPU^(^^17)^	34.5	8.4	0.23	81% *S* and >91% *σ* retention at 100% strain; 10+ stretch cycles without residual strain; dispersion stable >3 months	[93]
	[EMIM:DCA]	SiO_2_	14.8	47.5	0.21	*σ* measured from 25 to 90°C	[102]
	[EMIM:DCA]	PVDF-HFP (ethanol treated)	25.4	17.6	0.19	*T*_m_ ≈ 137°C; stable performance at 70%–75% RH	[88]
	[EMIM:DCA]	BC^(^^18)^	18.0	28.8	0.21	Thermally stable gel state (<85°C)	[96]
	[EMIM:DCA]	PU^(19)^	25.6	12.8	0.24	Stable TE performance at 50% strain; 95% strength recovery after self-healing; repeatable i-TE cycles after mechanical damage	[94]
	[EMIM:DCA]	Gelatin	2.83	22.9	0.47	Non-frozen down to −20°C; performance retention after 25 cycles (at 80°C): 8.6% for energy density and 31.4% for maximum output current	[100]
	[EMIM:DCA]	ZIF-8^(^^20)^	22.4	46.3	0.222	*σ* measured from −10°C to 70°C	[101]
	[EMIM:DCA]/ NaDCA	PVDF-HFP^(^^21)^	43.8	19.4	0.18	Increased *T*_g_ and gel-sol transition to −33.8°C and 134°C; stable energy output over 10 thermal cycles	[142]
	[EMIM:DCA]/ NaTFSI	PVDF-HFP	22.9	17.5	0.136	Thermally stable (<220°C); ∼100% *S* retention and 15% resistance change after 1000 bending cycles	[89]
	[EMIM:Ac]	PEO	18	1.1	–	Disintegrates at 80% RH; 300% elongation; excessive IL (>80 wt%) causes leakage and slow response	[95]
	[EMIM:OAC]	PEO/P123^(^^22)^	18	1.1	–	Stable *S* at 60% RH (over 80% retention after 600 h, or under up to 120% strain); disintegrates at 80% RH; 787% stretchability	[95]
	[EMIM:OAC]/ CH_3_COONa	Gelatin	37.3	12.3	0.24	Stable *S* over 15 cycles; increased *T*_g_ and *T*_m1_ to 33°C and 135.3°C; flexible but tensile strength drops significantly > 40°C	[98]
	[AMIM:Cl]/ZnCl_2_	Cellulose	−3.06	67.43	0.207	Thermally stable (−103–120°C); flexibility maintained 30 days at −80°C; 85% compressive recovery (5 cycles); repeatable 40-cycle i-TE performance	[97]
	[EMIM:TFSI]	PVDF-HFP/PEG	14.0	0.8	–	1-month shelf-life (25/35°C) with zero precipitation; 4-h continuous operation; reproducible over 5 thermal cycles	[58]
	[EMIM:TFSI]	P(MMA-co-MA)^(^^23)^/MXene	−8.8	3.18	0.19	*T*_g_ ∼ −10°C, thermally stable (<373 K); 1300%–2100% stretchability; <7% *S* and <10% *σ* fluctuation (20%–100% RH); waterproof; 120 s self-healing; 5 repeatable i-TE charging cycles	[99]
	[ChCl:EG]	WPU	19.5	8.4	0.20	Non-volatile; stable *S* > 1 month; ∼92% *S* retention at 50% strain; stable TE performance at 70%–90% RH	[333]
	[RMIM]Cl	PVA	10	1.6	0.2	Precursor shelf-life > months; *V*_oc_ stable >4 h; *S* differs depending on the metal electrode material	[92]
	[BMIM][BF_4_]	PVDF-HFP/AgOTf^(^^24)^	−26.4	4.2	0.18	Sol-gel transition 116.1°C; *T*_g_ ∼−22.3°C; stable *S* over 24 h	[90]
Polyelectrolytes	H^+^(PSSH^(^^25)^)	PSS^−^	7.9	90.0	0.38	>24 h *V*_oc_ retention in stage Ⅲ; i-TE device being electrically charged and discharged (5000 cycles); performance scaling with RH (30%–70%)	[145]
	Ag^+^(PSSAg^(^^26)^)	PSS^−^	−2.1	≈16.0	0.40	Air-stable; flexible; failure via Ag dendrite bridging; *S* and *σ* highly RH dependent (30%–70% RH)	[112]
	Na^+^(PSSNa)	PSS^−^	4.0	11.8	0.49	Increased *S* and *σ* across 50%–100% RH	[146]
	nH^+^(S-PEEK^(27)^)	(C_19_H_11_O_6_S^−^)_n_	5.5	20.0	–	RH-sensitive	[332]
	nCl^−^(PDDAC)^(^^28)^	(C_8_H_16_N^+^)_n_	18.0	19.0	–	*S* and *σ* highly RH-dependent (50× *σ* increase 30%–70% RH)	[332]
	nH^+^(Nafion^(^^29)^)	(C_7_F_13_O_5_S^−^·C_2_F_4_)_n_	3.6	19.0	–	Chemically stable across pH 1–10; less hydrophilic with increasing temperature	[332]

^(1)^ Polyethylene Oxide; ^(2)^ Polyvinyl Alcohol; ^(3)^ Polyquaternium-10; ^(4)^ poly(2-Acrylamido-2-Methylpropane Sulfonic Acid); ^(5)^ poly(Sulfobetaine Acrylate); ^(6)^ 2,2,6,6-tetramethylpiperidine-1-oxyl radical (TEMPO)-oxidized carboxylated bacterial cellulose; ^(7)^ Polyacrylamide; ^(8)^ Sodium Carboxymethyl Cellulose; ^(9)^ polydopamine; ^(10)^ Carboxylated Carbon Nanotubes; ^(11)^ Polyaniline; ^(12)^ Sodium Alginate; ^(13)^ Polyethylene Glycol; ^(14)^ Poly(3,4-Ethylenedioxythiophene): Poly(styrenesulfonate); ^(15)^ Ureidopyrimidinone; ^(16)^ Poly(Acrylamide-co-2-Acrylamido-2-Methylpropane Sulfonic Acid); ^(17)^ Waterborne Polyurethane; ^(18)^ Bacterial Cellulose; ^(19)^ Polyurethane; ^(20)^ Zeolitic Imidazolate Framework-8; ^(21)^ Poly(vinylidene fluoride-hexafluoropropylene); ^(22)^ Poly(ethylene oxide)-Poly(propylene oxide)-Poly(ethylene oxide) Triblock Copolymer; ^(23)^ Poly(methyl methacrylate-co-methyl acrylate); ^(24)^ Silver Trifluoromethanesulfonate; ^(25)^ Poly(styrenesulfonic acid) Hydrate; ^(26)^ Poly(styrenesulfonate) Silver Salt; ^(27)^Sulfonated Polyether Ether Ketone; ^(28)^ Polydiallyldimethylammonium Chloride; and ^(29)^ Perfluorosulfonic acid-PTFE copolymer.

Other denotations for column stability: TENG, triboelectric nanogenerator; *V*_oc_, open-circuit voltage; *C*_s_, specific capacitance; and *T*_m_, melting point.

#### Simple ions

**Figure 4.**
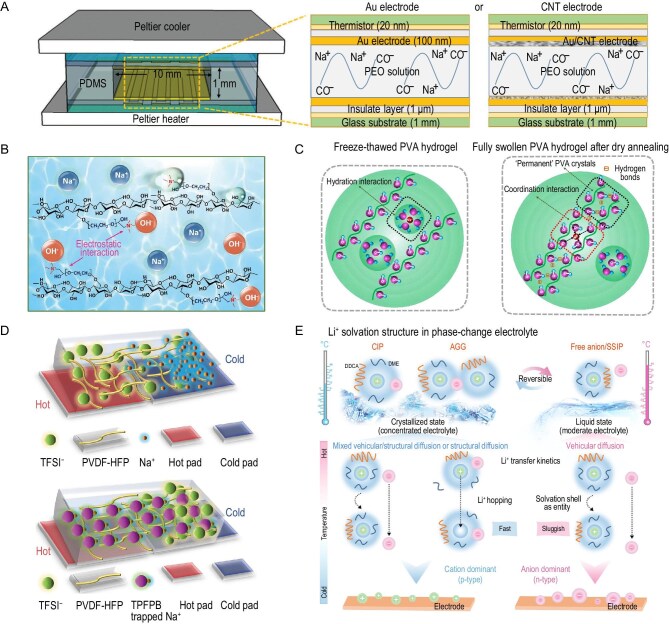
Simple ions for thermodiffusion. (A) Sketch of the i-TE supercapacitor device and the reaction that takes place in the solution. Reproduced with permission from Ref. [36]. Copyright © 2016 The Royal Society of Chemistry (RSC). (B) Schematic of the electrostatic interactions between PQ-10 and OH^−^ anions in the developed i-TE hydrogel. Reproduced from Ref. [135] under CC BY 4.0 license. Copyright © 2023 The Author(s). Published by Wiley-VCH GmbH. (C) Schematic of the ionic hydrogel of PVA and NaOH with synergistic coordination and hydration interactions. Reproduced from Ref. [136] under the terms of the CC BY-NC 4.0. Copyright © 2021 The Author(s), exclusive licensee AAAS. (D) The schematic of ion transport of p-type and n-type i-TE materials. Reproduced from Ref. [137] under CC BY 4.0 license. Copyright © 2022 The Author(s). (E) Design principle of the phase-change electrolyte for TDCs. Reproduced with permission from Ref. [138]. Copyright © 2025 RSC.

The initial exploration of i-TE materials focused on the thermodiffusion properties of simple ions, primarily derived from inorganic salts, acids, and bases, in early studies [11]. However, a fundamentally chemical limitation of these simple-ion systems is the insufficient mobility difference between cations and anions, which inherently constrains the achievable thermopower. To overcome this challenge, researchers have strategically engineered polymer–ion interactions to modulate ion transport and amplify mobility disparities selectively. A landmark study by Zhao *et al*. [36] in 2016 developed a PEO-NaOH i-TE system with a high thermopower of 10 mV K^−1^ through the TD effect (Fig. 4A). Subsequently, Li *et al*. [119] leveraged aligned cellulose nanochannels derived from oxidized natural wood, where the enhanced surface negative groups facilitated Na⁺ transport while impeding OH^−^ migration, yielding a thermopower of 24 mV K^−1^. Han *et al*. [135] utilized the cationic polymer polyquaternium-10 (PQ-10) as a host matrix, where quaternary ammonium groups selectively attracted OH⁻ and repelled Na⁺, enabling a thermopower of 24.2 mV K^−1^ (Fig. 4B). In a distinct strategy, Chen *et al*. [136] applied a dry-annealing process to coordinate Na⁺ with PVA, thereby promoting OH⁻ migration and achieving a significant negative thermopower of −37.6 mV K^−1^ (Fig. 4C).

Complementary to polymer-based modifications, molecular doping has emerged as an effective strategy for tuning i-TE properties. For example, Chi *et al.* [137] incorporated tris(pentafluorophenyl)borane (TPFPB) into a PVDF-HFP/propylene carbonate (PC)-Na:TFSI system, which suppressed Na⁺ transport and enabled reversible p–n switching, evidenced by a thermopower shift from +20 to −6 mV K^−1^ (Fig. 4D). Additionally, Xu *et al*. [138] developed a phase-change i-TE material comprising 1,2-dimethoxyethane (DME) and dimethyl dodecanedioate (DDCA). By precisely tuning the DME/DDCA ratio, they induced reversible crystalline-to-liquid phase transitions, transforming the Li⁺ transport mechanism from hopping to diffusion and resulting in a thermopower shift from +3.2 to −2.1 mV K^−1^ (Fig. 4E). Despite these significant advances, a comprehensive molecular-scale understanding of ion–polymer interactions underpinning both doping and polymer-based strategies remains elusive. Recent computational work by Chen *et al*. addressed this gap through molecular dynamics simulations of ether oxygen group coordination in PEO with metal cations. Their findings revealed that divalent ions exhibit markedly stronger coordination with PEO compared to monovalent ions, leading to significantly larger disparities in diffusion coefficients and consequently higher thermopower [139]. This highlights the potential of molecular-level design principles to optimize simple-ion i-TE systems in the future further.

#### Complex ions

The pursuit of high-performance i-TE materials demands further approaches to overcome the fundamental limitations of conventional simple-ion systems. Complex-ion architectures, which encompass tailored ILs, PEs, and their hybrid composites, offer unparalleled design flexibility to decouple and optimize ion transport properties. By engineering interactions between multiple ion species, polymer matrices, and nanoscale components, these systems enable precise control over thermopower, ionic conductivity, and environmental stability.

ILs have been recently utilized in thermodiffusion-type i-TE materials due to their low volatility, high thermal stability, and tunable properties [134]. ILs are defined as molten salts that exist in a stable liquid state at or near room temperature (typically below 100°C), composed entirely of discrete cations and anions. Unlike traditional simple salts (for example, NaCl and NaOH), which require high temperatures to melt, ILs maintain a liquid phase under ambient conditions due to their asymmetric ion structures and weak intermolecular forces. However, their practical application is constrained by inherently low thermopower, primarily stemming from insufficient mobility differences between cations and anions. To surmount this challenge, diverse chemical modulation strategies have been developed to optimize ion transport behavior and enhance thermoelectric performance.

**Figure 5.**
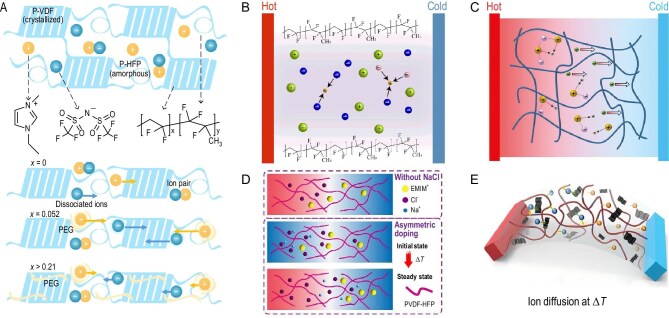
Complex ions for thermodiffusion. (A) Composition of PVDF-HFP-EMIM:TFSI i-TE gel and sketch of the interaction between ions, PEG molecules, and polymer matrix. Reproduced from Ref. [140] under CC BY 4.0 license. (B) Schematic to illustrate the interaction between Li^+^ cations and anions during the thermodiffusion of ions in the polymer channel. Reproduced from Ref. [141] under CC BY-NC 4.0 license. Copyright © 2022 The Author(s), exclusive licensee AAAS. (C) Schematic illustration of the effect of AgOTf on the PB ionogels. Reproduced with permission from Ref. [90]. Copyright © 2023 Wiley-VCH GmbH. (D) Directional ion diffusion induced by asymmetric ion doping. Reproduced from Ref. [143]. Copyright © 2025 ACS. (E) Schematic illustration of the ion thermodiffusion in the ionogel under a temperature difference. Reproduced from Ref. [99] under CC BY 4.0 license. Copyright © 2023 The Author(s). Published by Wiley-VCH GmbH.

One prominent approach is to introduce polar groups in polymer hosts that selectively interact with IL ions, thereby facilitating preferential ion migration. A typical example is the PVDF-HFP/EMIM:TFSI system reported by Zhao *et al*., where PEG was incorporated to form hydrogen bonds with EMIM⁺ ions, thereby promoting cation diffusion and tuning the thermopower from −4 to 14 mV K^−1^ (Fig. 5A) [140]. Ion-selective doping represents another effective strategy for enhancing thermopower. By incorporating additional ions to regulate cations and anions mobility disparities, Liu *et al*. achieved a tunable thermopower ranging from −15 to +17 mV K^−1^ in PVDF-HFP–EMIM:TFSI through the addition of LiBF_4_ or EMIM: Cl (Fig. 5B) [141]. Similarly, Ouyang *et al*. employed Na⁺ doping to strongly interact with DCA⁻, suppressing anion transport and boosting the thermopower of PVDF-HFP/EMIM:DCA to 43.8 mV K^−1^ [142]. Le *et al*. further demonstrated controllable p–n type switching by doping AgOTf, achieving a transition from +5.5 mV K^−1^ to −26.4 mV K^−1^ (Fig. 5C) [90]. Additionally, Zhao *et al*. introduced NaCl into EMIM:Cl to establish an internal NaCl concentration gradient, effectively modulating ion diffusion and increasing thermopower from 24.4 to 40.3 mV K^−1^ (Fig. 5D) [143]. Moreover, the incorporation of nanofillers or composite components has also proven instrumental in improving ion transport and environmental stability. For instance, Zhao *et al*. [99] introduced MXene nanosheets into an EMIM:TFSI-based gel. The negatively charged surface groups of MXene interacted with EMIM⁺ cations, thereby reinforcing the n-type thermopower of −8.8 mV K^−1^ and improving ambient stability (Fig. 5E). In another study, Yin *et al*. [143,144] designed a quaternary ionic gel consisting of EMIM:TFSI, Na:TFSI, and PEG. By utilizing the synergistic effect of Coulombic interaction between Na⁺ and TFSI⁻ and hydrogen bonding between PEG and EMIM⁺, it achieved a high thermopower of 21.2 mV K^−1^.

The PEs typically exhibit slower ion migration rates but possess significantly larger cation–anion mobility disparities, enabling a higher thermopower potential [14]. In 2016, Kim *et al*. [145] demonstrated that poly(styrenesulfonic acid) hydrate (PSSH) thin films generated a thermovoltage under a temperature gradient via the TD effect, with a thermopower reaching 7.9 mV K^−1^ at 70% relative humidity (RH). Subsequently, Wang *et al*. [146] elucidated the critical role of humidity in i-TE performance, reporting a thermopower of 4 mV K^−1^ in PSSNa films at RH = 100%. Collectively, these strategies highlight that despite the intrinsic challenges of these complex ions, ILs and PEs, their thermoelectric performance can be substantially improved through rational molecular design and optimization of ion–polymer interactions, particularly by amplifying ion mobility differences and stabilizing ion transport pathways.

### Choice of thermogalvanic redox couples

#### Conventional redox couples

To date, the p-type [Fe(CN)_6_] ^4^⁻^/3^⁻ and n-type Fe^2+/3+^, I^−^/I_3_^−^ redox couples are among the most extensively studied, owing to their favorable electrochemical properties and accessibility. Multiple strategies have been employed to enhance thermopower by increasing both reaction entropy and concentration differences, considering the specific chemical characteristics of each system. These approaches have established general principles for tuning redox reactions to efficiently harvest low-grade heat.

**Figure 6.**
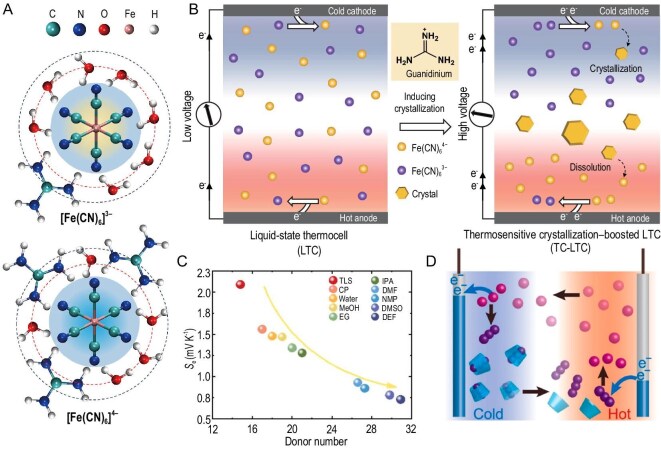
Commonly used redox couples and representative strategies for thermopower enhancement. (A) Enhancement of reaction entropy in [Fe(CN)_6_]^4^⁻^/3^⁻ via Gdm^+^ interaction. Reproduced from Ref. [66] under CC BY 4.0. Copyright © 2018, The Author(s). (B) Temperature-sensitive crystallization induced by Gdm^+^ and Fe(CN)_6_^4^⁻. Reproduced with permission from Ref. [38]. Copyright © 2020 The Author(s), exclusive licensee AAAS. (C) Increase in thermopower of Fe^2+/3+^ redox couple through modulation of the solvation environment with organic solvents. Reproduced with permission from Ref. [152]. Copyright © 2022 RSC. (D) Selective capture of triiodide ions by α-cyclodextrin at low temperatures. Reproduced with permission from Ref. [42]. Copyright © 2016 ACS.

[Fe(CN)_6_]^4^⁻^/3^⁻: Ferricyanide/ferrocyanide ions are commonly used for electrochemical calibration, which was firstly reported by Brian Burrows in 1976, owing to their highly reversible and ultrafast reaction kinetics. Their intrinsic thermopower of 1.4 mV K^−1^ in 0.4 M aqueous solution highlights substantial potential for harvesting low-grade heat. To further enhance thermopower, modifications in solvation structure [147], such as the use of organic solvents, have proven effective. For instance, thermopower rises from 1.4 to 2.9 mV K^−1^ in a methanol–water mixture due to solvation shell restructuring [71]. Additionally, specific counterions can perturb the solvation environment [148]; for example, guanidinium (Gdm^+^) ions, through strong chaotropic interactions, boost thermopower to 4.2 mV K^−1^ (Fig. 6A) [66]. Gdm^+^ also induces thermosensitive crystallization with Fe(CN)_6_^4−^, amplifying ${\mathrm{\Delta }}C$ between electrodes (Fig. 6B) [38]. Thanks to its high thermopower and robust chemical stability, this redox couple is featured in diverse thermogalvanic applications. Advances in materials and device engineering have enabled high energy conversion efficiencies, compatibility with hydrogels [74], and broad operational temperature windows [149], positioning this system as a prime candidate for both energy harvesting and self-powered devices.

Fe^2+/3+^: For ferric/ferrous ions coordinated by hexa-aquo ligands, [Fe(H_2_O)_6_]^2+/3+^, the ionic charge exerts a significant influence on the reaction entropy. In acidic solutions, suppression of Fe^3^⁺ deprotonation leads to fewer [Fe(OH) (H_2_O)_5_]^2^⁺ species with reduced charge, thereby enhancing the thermopower of this redox couple [150]. The presence of counter ions also modulates the net charge of iron ions. For example, a higher thermopower of −1.66 mV K^−1^ is observed with perchlorate anions, due to their non-coordinating nature, whereas chloride anions, which tend to form [Fe(H_2_O)_5_Cl]^+/2+^ complexes with iron, reduce the total charge and yield a lower thermopower of −1.04 mV K^−1^ [151]. Recent studies have also investigated the effect of organic solvents on the solvation structure around Fe^2^⁺/^3^⁺ ions to modulate reaction entropy. For instance, the use of tetramethylene sulfone, an organic solvent with a low donor number, results in a more compact solvation shell around Fe^3^⁺, increasing the thermopower to −2.49 mV K^−1^ (Fig. 6C) [152]. Similarly, the addition of acetonitrile (MeCN) enhances the thermopower to −3.73 mV K^−1^ by selectively interacting with Fe^2^⁺ ions and reorganizing the dipole orientation of surrounding solvent molecules [153].

I^−^/I_3_^−^: The iodide/triiodide redox couple exhibits pronounced differences in hydrophilicity between its components. While iodide ions are highly hydrophilic, triiodide ions are significantly more hydrophobic, resulting in selective binding affinities for specific polymers and supramolecular hosts. Notably, temperature-dependent interactions between triiodide ions and host molecules can establish a concentration gradient across the TGC, thereby markedly enhancing thermopower. For example, temperature-sensitive host–guest complexation between triiodide ions and α-cyclodextrin (α-CD) supramolecules can generate a triiodide concentration gradient, leading to an increased thermopower of −1.9 mV K^−1^ for the I^−^/I_3_^−^ redox couple (Fig. 6D) [42]. Thermo-responsive hydrogels, such as poly(N-isopropylacrylamide) (PNIPAM), become hydrophobic at their phase transition temperature and are capable of selectively capturing triiodide at higher temperatures, even reversing the sign of thermopower [58]. Similarly, Han *et al*. introduced thermally responsive MC to promote hydrophobic interactions with triiodides, enabling n–p conversion of thermopower above the gelation temperature through tuning of both the concentration gradient and reaction entropy [83]. In this context, materials exhibiting temperature responsiveness and phase transitions have become important strategies for tuning the thermopower of the I^−^/I_3_^−^ system [59,154].

#### Finding novel redox couples

Beyond iron-based redox couples, a variety of other metal ions have been explored for their chemical stability and promising thermoelectric properties. The intrinsic thermopower of several non-iron metal redox systems has been reported, including [Ni(bpy)_3_]^2+/3+^ (−2.8 mV K^−1^) [155], [Co(bpy)_3_]^2+/3+^ (−1.5 mV K^−1^) [156], [Ru(bpy)_3_]^2+/3+^ (−0.5 mV K^−1^), Cu/CuCl_2_ (−0.7 mV K^−1^) [157], and V^2+/3+^ (−1.7 mV K^−1^) [158]. The relatively high thermopower in these systems is mainly attributed to ligand interactions and spin-state transitions [45,54]. Strategies for enhancing thermopower, such as tuning the solvation environment, have also been successfully applied to these systems; for example, modifying solvent interactions around [Co(bpy)_3_]^2+/3+^ in ILs increases its thermopower from −1.5 to −2.2 mV K^−1^ [54].

Organic redox couples have attracted considerable interest in recent years due to their derivation from biomaterials and unique proton-coupled electron transfer (PCET) mechanisms. In PCET systems, electron transfer is inherently linked with proton transfer, leading to substantial entropy changes and consequently elevated thermopower. For example, the oxidation of [Ru^II^(Him)_5_(im)]^+^ to [Ru^III^(Him)_2_(im)_4_]⁻ in a pH 10.9 solution involves the release of three protons, resulting in a thermopower of 2.7 mV K^−1^, significantly higher than the –0.8 mV K^−1^ measured in buffer solutions with the pH ranging from 2 to 8.6 where proton involvement is minimal [159].

Quinones represent a diverse class of organic redox couples, with extensively studied derivatives such as hydroquinone (HQ) and anthraquinone-2,7-disulfonic acid [160–163]. Notably, Guo *et al*. leveraged the phase-transition properties of nanogels to generate a pH gradient, driving a PCET process in HQ and achieving a peak thermopower of 6.7 mV K^−1^ near the phase transition temperature [160]. Beyond quinones, other organic redox couples are under active investigation. Methyl viologen exhibits a temperature coefficient of 0.6 mV K^−1^ [158], while pyrazine-based redox species can deliver thermopower exceeding 3 mV K^−1^ [164]. The temperature sensitivity and tunability of these organic redox systems make them particularly promising for integration into i-TE device that pursuing efficient low-grade heat harvesting.

#### Engineering the hydro-structure of redox couples

The hydration structure of ions plays a crucial role in i-TEs, but its influence appears in distinct ways depending on the mechanism. In redox couples, the decisive factor is the solvation entropy difference between oxidized and reduced states, which sets the reaction entropy and governs the temperature dependence of electrode potentials [34]. In contrast, hydration shells primarily affect ion mobility and concentration gradients under a thermal field in thermodiffusion systems [165]. While the thermodynamic expressions differ, both cases underscore the importance of ion–solvent interactions as a factor of thermopower. Strongly hydrated ions, such as Fe^3^⁺, impose a rigid orientational ordering on surrounding water molecules, leading to lower solvation entropy compared to their reduced counterparts such as Fe^2^⁺. The greater the contrast between hydration environments, the steeper the temperature coefficient of electrode potential. Classical aqueous redox couples such as Fe^2+/3^⁺ or [Fe(CN)_6_]^4−/3−^ deliver modest thermopowers of about 1–2 mV K^−1^.

**Figure 7.**
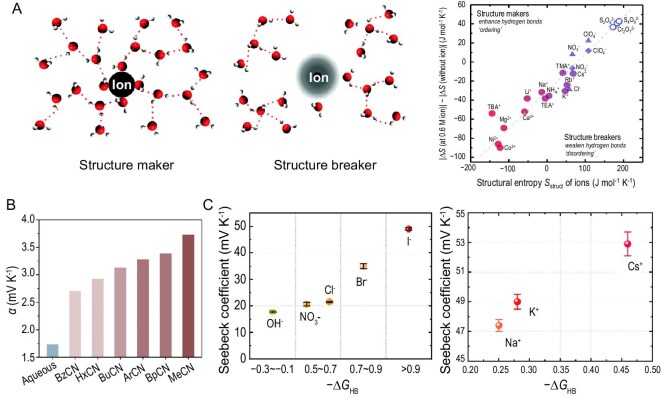
Hydration structure effects on thermopower. (A) Change of redox reaction entropy of hydrated Fe^2+/3+^ after adding supporting ions. Adapted from Ref. [44] under CC BY 3.0 license. (B) Switching solvent from water to non-aqueous solution to improve *S*_TG_. Reproduced with permission from Ref. [153]. Copyright © 2025 Elsevier Inc. (C) Relationship between the thermopower and the −Δ*G*_HB_ values of the anions and cations. Reproduced with permission from Ref. [168]. Copyright © 2022 ACS.

Recent advances have shown that the purposeful modulation of solvation shells by introducing non-aqueous solvents or electrolyte additives can markedly improve performance. Inoue *et al*. [166] demonstrated that switching the solvent from water to acetone increased the solvation entropy difference and achieved thermopowers up to 3.6 mV K^−1^. Huang *et al*. [44] revealed that adding redox-inert supporting electrolytes could enhance ∆*S*_sol_ and thus the thermopower by altering the hydrogen-bond network of water (Fig. 7A). They attributed this effect to the structure-making and structure-breaking roles of supporting ions: the former strengthens the hydration network and increases ordering, while the latter weakens interactions and induces disorder in the solvation shell. Building on these concepts, Zeng *et al*. [153] combined these two ways by introducing weakly coordinating anions (ClO_4_⁻) and polar cosolvents (MeCN) to alter the hydration structure of Fe^2^⁺ relative to Fe^3+^ selectively. This strategy effectively enlarged the solvation entropy gap, increasing the thermopower to nearly 4 mV K^−1^ (Fig. 7B).

On the contrary, the underlying physics of thermodiffusive thermopower can be directly traced to Eastman entropy of transfer, which quantifies the heat carried per ion during migration, and is strongly influenced by whether ions act as ‘structure makers’ or ‘structure breakers’ in the solvent network [34]. Structure-making ions, such as large quaternary ammonium cations, reinforce hydrogen-bond networks and stabilize extended solvation shells, leading to enhanced order compared to the bulk liquid. This stabilization lowers solvation entropy but amplifies the entropic contrast that drives strong thermodiffusion, as exemplified by tetrabutylammonium nitrate, resulting in thermopower up to 7 mV K^−1^ in alcohol solvents [34]. By contrast, structure-breaking ions disrupt solvent–solvent interactions, thinning the solvation shell and creating local disorder. Such ions often promote greater mobility asymmetry between cations and anions, which can also enhance thermodiffusive thermopower under closed-boundary conditions.

Recent studies further demonstrate that the Gibbs free energy of transfer associated with hydrogen-bond rearrangement (∆*G*_HB_) provides a quantitative measure of this effect [167]. A more negative ∆*G*_HB_ corresponds to stronger structure breaking, which has been shown to increase the ionic thermopower in polymer electrolytes and gels (Fig. 7C). For example, in PVA hydrogels, both cations and anions with strong structure-breaking character enhanced thermodiffusion by altering ion–polymer interactions and promoting asymmetric drift [168]. These insights highlight that, beyond size or charge density, the role of ions as structure makers or breakers is a decisive factor in governing entropy transport and thus the magnitude of thermodiffusion in diverse electrolyte systems.

The concentration ratio difference is also a key factor determining the performance of i-TEs. In i-TE generators based on the thermogalvanic effect, the concentration ratio differences between the redox couples will also affect the thermopower by influencing the redox reaction rates. Therefore, engineering the concentration ratio differences between ions plays a key role in tailoring the thermopower in thermogalvanic i-TE generators. It is important to consider the water shell of the ions when exploring i-TE hydrogels or solutions [169]. Therefore, engineering the hydro-structure of the ions to tailor the concentration ratio differences of ions can ultimately impact the thermoelectric output.

The thermopower of the thermogalvanic-dominant mechanism is also strongly related to the concentration ratios. As described by Nernst equation, the equilibrium potential *E* of the redox reaction can be expressed as


(4) \begin{eqnarray*}
E = {E}_0 + \frac{{RT}}{{nF}}\left[ {\ln \frac{{{\gamma }_{{\mathrm{ox}}}}}{{{\gamma }_{{\mathrm{red}}}}} + \ln \frac{{{C}_{{\mathrm{ox}}}}}{{{C}_{{\mathrm{red}}}}}} \right],{\mathrm{\ }}
\end{eqnarray*}

where *E* is potential, ${E}_0$ is standard potential, *R* is the ideal gas constant, *T* is the temperature, *n* is the number of electrons moved in the electrochemical process, and *F* is the Faraday constant, respectively [11]. The key factors that determine the potential should be the activity coefficient ($\gamma $) and concentration (*C*) of redox ions. The thermopower can be calculated by the ratio of the equilibrium potential difference between the hot and cold ends to the temperature difference. Therefore, from this equation, the thermopower of the TGCs should be strongly related to the concentration ratio difference of the redox ions. By utilizing the thermosensitive guanidinium cations (Gdm^+^) to modify the concentration ratio difference of the redox ions ([Fe(CN)_6_]^4−/3−^), the thermopower of the TGCs can be boosted from 1.4 to 3.73 mV K^−1^ [38]. Similar strategies can also be applied in I^−^/I_3_^−^ and Cu/Cu^2+^ TGCs [59,60]. Moreover, by utilizing the differences in the solubility of redox couple ions in solvents and the differences in the influence of temperature on ion solubility, some studies have modulated the solvent to enhance the concentration ratio of redox couple ions in local regions, thereby improving the thermopower [153,170]. Furthermore, utilizing ion–polymer interactions can also change the concentration ratio difference and thus increase the thermopower of the TGCs [42].

### Designing the polymer networks

Polymer provides a new dimension to engineer the local environment of the thermodiffusion ions and thermogalvanic ionic couples in the i-TE materials, including all of the forms of liquid, gel, PE, and porous solid. The polymer network serves not only as the structural framework that reshapes ionic transport channels but also contributes to the macroscopic morphological stability of the materials, due to its excellent mechanical properties, playing a crucial role in regulating device performance [11,171].

#### Simple polymers

**Figure 8.**
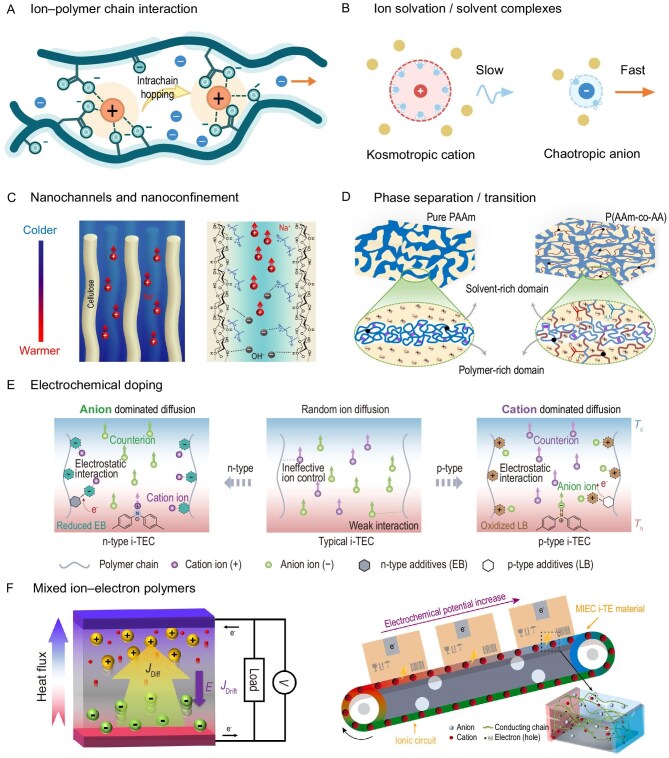
Schematic diagram of polymer design mechanisms. (A) Schematic illustration of ion–polymer chain interactions and transport processes in the PEO-Li^+^ system, where coordination sites facilitate intrachain hopping [174]. Moreover, the ion–polymer interaction acts as a selective filter, slackening, or hindering the transport of specific ions while facilitating the thermodiffusion of counter-ions. This mechanism significantly amplifies the thermodiffusion discrepancy between cations and anions. (B) Ion–solvent interactions significantly dictate the ion solvation structure. Typically, kosmotropic cations with large hydrated radii exhibit lower mobility, whereas chaotropic anions with smaller hydrated radii demonstrate faster transport. Furthermore, specific ion-solvent systems can form stable complexes, such as in the PAAm-Fe^2+/3+^ material [176], which modulate the energy barrier and transport kinetics of thermodiffusion. (C) Schematic of the enhanced ion mobility and selectivity of the cellulosic membrane due to the nanochannels formed between the cellulose nanofibers and nanoconfinement effect. Reproduced with permission from Ref. [119]. Copyright © 2019, The Author(s), under exclusive licence to Springer Nature Limited. (D) Phase separation leads to the formation of distinct polymer-rich and solvent-rich phases. Reproduced with permission from Ref. [196]. Copyright © 2022, The Author(s), under exclusive licence to Springer Nature Limited. Notably, phase transitions can drastically alter physical characteristics (for example, viscosity) and ionic transport dynamics across the temperature gradient, thereby amplifying thermoelectric performance [319,320]. (E) Ion transport control in i-TE cells engineered by electrochemical doping for p-type and n-type systems, respectively. Reproduced from Ref. [197] under CC BY-NC-ND 4.0 license. Copyright © 2026 the Author(s). Published by The Proceedings of the National Academy of Sciences (PNAS). (F) Ionic–electronic dual-conduction mechanism. Reproduced from Ref. [209] under CC BY 4.0 license and with permission from Ref. [321] (Copyright © 2024 Elsevier Inc.).

Conventional simple polymers are macromolecular substances formed by the polymerization of identical or similar monomers, exhibiting either linear or branched structures with homogeneous chemical composition. Typical examples include PEO, PVA, PAAm, and polyacrylonitrile (PAN), among others [172]. Ion transport within these polymers primarily occurs via two mechanisms, one of which facilitated by segmental motion of the polymer chains, and the other enabled by solvation within the polymer matrix [173]. A representative example of segmental-motion-assisted ion migration is the PEO-based polymer composite, where lithium ions coordinate with ether oxygen atoms and migrate along the polymer chain between coordination sites [174], as illustrated in Fig. 8A. In contrast, gel polymer i-TE materials rely heavily on solvation [175], where the ions form ion–solvent complexes with the liquid medium or functional groups in the polymer matrix, as illustrated in Fig. 8B [176]. The gel matrix stabilizes the ions, lowers the energy barrier for migration, and enhances both diffusion efficiency and ionic conductivity. Moreover, the coupled dynamics of solvent molecules and polymer chain flexibility further promote ion transport [176,177].

Modulating ion–polymer interactions has emerged as an effective strategy for enhancing thermopower [136]. For instance, Zhao *et al*. [36] incorporated NaOH into PEO to convert terminal hydroxyl groups (–OH) into alkoxide end groups (–O–Na⁺). The resulting polycations served as mobile carriers, improving ion transport rates and achieving a high thermopower of 10 mV K^−1^. In another study, Yu *et al*. [178] developed an n-type i-TE material based on PVA and CuCl_2_, and the hydrophilic hydroxyl groups of PVA coordinated with Cu^2+^, enhanced the thermodiffusion disparity between Cl⁻ and cations and yielded a thermopower of –18.1 mV K^−1^.

Alternatively, tuning the solvation environment offers another promising route toward higher thermopower [179,180]. For example, Wang *et al*. [179] designed an n-type i-TE material comprising PEO, lithium salts, and an IL. The strong coordination between the ether oxygen atoms and lithium ions reinforced the solvation structure, while preferential ion aggregation led to anion-rich clusters that modified the solvation environment. This optimization in anion transport resulted in a thermopower of –15 mV K^−1^. Du *et al*. [181] developed an i-TE material based on a PVDF-HFP matrix, a mixed solvent of dimethyl carbonate (DMC) and ethylene carbonate (EC), and a lithium salt. In this system, the weak coordinating interaction between fluorine atoms in PVDF-HFP and Li⁺ ions was leveraged, while the polarity difference between DMC and EC was utilized to modulate the ion solvation structure. By optimizing the lithium salt concentration and solvent ratio, the authors suppressed ion aggregation and promoted the generation of free ions, thereby modifying the solvation environment of Li⁺. This optimization enhanced the directional migration efficiency of Li⁺ under a temperature gradient, ultimately yielding a high thermopower of 17 mV K^−1^ [181].

#### Complex polymers

Although simple polymers can achieve high thermopower, their networks often suffer from mechanical weaknesses, such as low strength and a tendency toward fracture deformation, which stem from monotonous internal structures and weak intermolecular interactions [182,183]. Furthermore, the absence of ordered ion transport channels leads to low thermoelectric conversion efficiency and suboptimal thermopower, rendering these key parameters inadequate for practical applications [4]. Recent advances in complex polymer networks have been achieved by constructing multi-level cross-linked architectures, which significantly enhance mechanical properties [184]. Through optimized polymer–ion interactions, researchers have improved thermoelectric conversion efficiency while integrating functionalities such as self-healing and stimulus-responsive behavior, effectively overcoming the constraints of conventional polymer networks.

Complex polymer networks can enhance i-TE performance by modifying the morphology and surface functional groups of simple polymer segments [185–188], thereby enhancing ionic transport. For example, Li *et al*. [119] transformed hydroxyl groups (–OH) into carboxyl groups (–COOH) on cellulose molecular chains, altering the ionic transport environment and generating negatively charged nanocellulose channels (Fig. 8C). This modification enabled more efficient sodium ion migration under thermal gradients, achieving a thermopower of 24 mV K^−1^, which is significantly higher than that of the unmodified cellulose polymers [119]. Additionally, tuning the spatial arrangement of polymer segments can also provide selective ion transport pathways [87,140,145]. Chen *et al*. [136] employed dry annealing to promote hydrogen bond-driven self-assembly among PVA chains, aligning them into oriented crystalline structures. This process created microscale directional nanochannels that facilitated the migration of cationic Na⁺ while repelling anionic OH⁻, resulting in a high thermopower of –37.61 mV K^−1^. Similarly, Meng *et al*. [189] utilized controlled drying to induce phase separation of LiTFSI within a polymer matrix, resulting in the formation of ion-rich and ion-depleted regions. This heterogeneity established continuous 3D ionic channels that supported selective cation migration over anions under a temperature gradient, yielding a thermopower of 25 mV K^−1^.

Furthermore, the construction of dual or multi-network architectures via chemical cross-linking is an effective strategy for enhancing both mechanical and self-healing properties [4,51,190,191]. A common approach involves sequentially cross-linking a single homopolymer to form dual-network polymers. For example, Wu *et al*. [184] polymerized a concentrated acrylamide (AM) solution to create the primary network for stiffness, then immersed it in a dilute AM solution to form the secondary network for extensibility. This dual-chemical-crosslinking network polymer is capable of accommodating high-concentration electrolytes while exhibiting exceptional mechanical performance (217% elongation at break, 1190 kPa tensile strength).

Moreover, copolymerizing distinct monomers further enhances the mechanical properties of polymer-based i-TE materials [192–195]. In contrast to Wu’s two-step method, Hu *et al*. [196] adopted a simpler one-step copolymerization of acrylamide and acrylic acid in 1-ethyl-3-methylimidazole ethyl sulfate. This process formed a macroscopically uniform covalent network with phase separation, comprising a hydrogen-bond-rich polymer phase that provides strength and a solvent phase that confers elasticity (Fig. 8D). The resulting material demonstrated a tensile strength of 12.6 MPa, substantially exceeding those of pure PAAm (2.2 MPa) and PAA (3 MPa), while the hydrogen-bonding network also imparted self-healing capabilities [196]. Furthermore, mechanical tensile reinforcement also serves as an effective strategy for enhancing material properties. Ma *et al*. [74] showed that repeated freeze-thaw cycles of PVA under a constant 200% tensile strain induced chain crystallization and the formation of layered anisotropic structures. This method significantly enhanced mechanical properties, achieving an exceptional tensile elongation of 1300%—far surpassing conventional polyacrylamide-based hydrogels (typically 400%–800%)—while maintaining a high thermopower of 6.5 mV K^−1^.

More recently, Li *et al*. [197] established a fundamentally new framework for electrolyte design through electrochemical doping. Rather than treating electrolytes as passive media in which cations and anions migrate concurrently, the authors introduced electroactive polymers (that is, leucoemeraldine base and emeraldine base) as dynamically addressable ionic regulators to achieve ion transport control. As illustrated in Fig. 8E, these polymers selectively coordinate with ions (that is, Cl^−^ or K^+^) in i-TE cells, effectively immobilizing one ionic species while leaving the other as the dominant charge carrier. Mechanistically, the redox-state transition of the polymer backbone modulates specific ion–polymer coordination interactions, thereby redistributing ionic species within the electrolyte. This selective ion confinement substantially alters transference behavior, leading to pronounced changes in ionic selectivity and transport efficiency. Importantly, the electrochemical doping process is reversible and bias-dependent, allowing temporal control over ion flux without altering bulk composition. When implemented in i-TE cell systems, this strategy can not only enhance the thermodiffusive asymmetry between cations and anions, but also facilitate confined redox reactions through a redox-coupled electron relay mechanism, underscoring its functional significance. Conceptually, the work reframes electrolyte engineering from static compositional tuning to electrochemically programmable ion management. By coupling redox chemistry with ion transport regulation, it builds a bridge between conducting polymer physics and electrolyte science, offering a generalizable pathway toward adaptive ionic conductors for energy harvesting, storage, and ionotronic devices.

#### Mixed ion–electron polymers

Currently, i-TE materials generally suffer from low energy density and low power output, primarily due to inherent limitations such as the ion migration rate and carrier concentration [66,122,198]. Among them, thermodiffusion-type i-TE materials face particular challenges in achieving continuous and stable energy output due to their reliance on capacitive charge/discharge mechanisms [92,199,200]. These issues severely restrict the practical implementation and widespread application of i-TE technology in low-grade heat recovery. The ion–electron coupled transport strategy offers a critical new pathway to address these challenges [36,201].

Existing studies have shown that PEDOT:PSS inherently exhibits dual conduction capabilities for both electrons and ions [202–206]. For instance, Kim *et al*. [207] utilized the metal coordination between CuCl_2_ and PEDOT:PSS to achieve n-type thermoelectric conversion through Cl⁻ transport in a conductive polymer system, successfully fabricating devices with a power factor as high as ∼1.7 mW m^−1^ K^−2^. However, it should be noted that such ion–electron conductive polymer matrices still suffer from relatively low conductivity. To address this limitation, further utilization of the internal ion–electron thermoelectric synergistic effect is required (Fig. 8F). The core of this approach lies in constructing a polymer matrix capable of simultaneous ion and electron conduction, wherein a temperature gradient drives ion thermodiffusion to generate a built-in electric field that directs electron drift, thereby enabling continuous current output [208–212]. For example, Ouyang *et al*. [208] developed a thermodiffusion-dominated i-TE device by constructing a conductive carbon scaffold impregnated with BMIM:Cl electrolyte combined with copper electrodes, which achieved a maximum power density of 0.23 W m^−2^ and an energy density of 12.5 J m^−2^. Building on this, Sun *et al*. [209] proposed an ion–electron thermoelectric synergy mechanism. Using carbonized pomelo peel as a carrier material, they designed a novel i-TE material that capitalizes on the combined contribution of electrons and ions, enabling sustained device operation. This material achieved a maximum power density of 0.41 W m^−2^ and an energy density of 553.9 J m^−2^, which is over 6.9 times higher than that of traditional i-TE materials. There are still other efforts to engineer the coupling effect between electrons and ions [178,212]. However, further separating the detailed contributions of ions and electrons remains a challenge.

A fundamental challenge in MIECs is the internal shunting effect, where the dominant electronic phase short-circuits the ion-induced thermovoltage. To decouple these pathways, three primary strategies have emerged: (i) structural-gradient engineering, which utilizes segregated networks—such as longitudinal water channels—to confine ionic transport while preserving a continuous electronic backbone [213]; (ii) interfacial energy-filtering, employing selective layers (for example, PSSH) to transfer ion-induced potential to high-energy electronic carriers, thereby amplifying *S* without compromising *σ*_e_ [214]; and (iii) morphology engineering, which creates hierarchical architectures that separate ionic and electronic transport length scales via porous/dense phase integration [215]. Crucially, a transformative paradigm of dynamic electrochemical regulation mentioned above has emerged (Fig. 8E), where the electronic phase is reframed from a passive parasitic shunt into a dynamically addressable ionic regulator. Mechanistically, the intrinsic electronic conductivity of the polymer backbone facilitates a redox-coupled electron relay; this process accelerates confined redox reactions by transferring external electrons rather than merely short-circuiting the thermovoltage. Consequently, this approach transforms the interaction between ionic and electronic carriers from an antagonistic relationship into a synergistic booster for energy conversion efficiency. Elucidating such synergistic coupling between thermodiffusive ions and electronic redox chemistry serves as an instructive strategy for overcoming the *σ*–*S* trade-off in advanced MIEC systems.

## CHALLENGES IN PHYSICS OF I-TE

### The transport of ions in i-TE

#### Ion size effect

Ion size is one of the primary factors affecting its transport behavior. The physical size of ions significantly alters their diffusion rate and diffusion path in electrolytes or matrices. First, considering that ion diffusion results from Brownian motion, the ion concentration in high-temperature areas is lower under a temperature gradient than in the low-temperature areas. Assuming that ions undergo three-dimensional Brownian motion in the solvent, Stokes–Einstein relations for the diffusion coefficient *D* of a spherical particle in a fluid can be presented as [216]


(5) \begin{eqnarray*}
D = \frac{{{k}_{\mathrm{B}}T}}{{{\mathrm{6}}\eta r}},{\mathrm{\ }}
\end{eqnarray*}

where ${k}_{\mathrm{B}}$, $\eta $, and *r* are the Boltzmann constant, viscosity, and particle radius, respectively. Generally, small-sized particles have lighter mass, lower inertia, weaker interaction with the matrix, lower migration energy barriers, and are more sensitive to temperature changes. They are more likely to move through diffusion or hopping mechanisms in confined spaces, thus exhibiting higher diffusion rates. Some large-sized ions also possess higher charge amounts than small-sized ions, and ion size can also affect their solvation behavior in electrolytes [175]. Small-sized ions such as Li⁺ have smaller solvation shell sizes, fewer surrounding molecular coordination sites, and less restriction on their diffusion degrees of freedom. In contrast, large-sized ions such as Mg^2+^ exhibit lower migration rates due to thicker solvation layers and a larger number of surrounding coordination molecules. The size dependence of ion transport is particularly significant in nano-confined structures, where confined spaces further amplify the influence of ion size on migration mechanisms. For example, small-size ions such as H^+^ may transport rapidly through the Grotthuss mechanism [217,218]. In contrast, ions with large effective radii or strong coordination interactions typically exhibit sluggish kinetics. This transport feature is evidenced in the PVDF-HFP-EMIM:DCA-Na:DCA system, where Na^+^ doping substitutes slower cationic species, thereby amplifying the net thermopower [142].

#### Ion concentration effect

**Figure 9.**
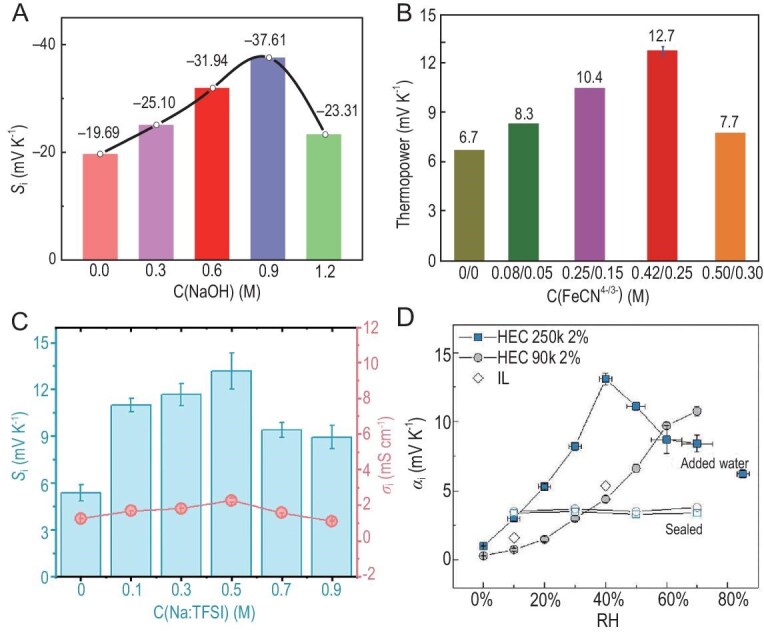
Ion concentration effect on thermopower. (A) Thermopower of PVA hydrogels with different concentrations of NaOH after dry-annealing. Adapted from Ref. [136] under CC BY-NC 4.0 license. Copyright © 2021 The Author(s), exclusive licensee AAAS. (B) Thermopower of Gelatin-0.8 M KCl-m/n FeCN^4–/3–^ i-TE gel. Adapted with permission from Ref. [4]. Copyright © 2020 The Author(s), exclusive licensee AAAS. (C) Thermopowers of ionogels with different Na:TFSI concentrations. Reproduced from Ref. [144] under CC BY 4.0 license. Copyright © 2024 The Author(s). Published by Innovation Press. (D) The effect of humidity (indirectly affecting ion concentration) for apparent thermopower of sealed and open electrolytes with HEC 250k, HEC 90k, and only IL. Adapted from Ref. [322] under CC BY 3.0 license.

Ion concentration is another key parameter, and its impact on i-TE performance manifests as a double-edged sword effect in recent study as shown in Fig. 9. On the one hand, increasing the ion concentration appropriately can enhance carrier density, thereby improving conductivity and thermopower. For example, as the salt concentration increases in liquid electrolytes, ion mobility usually increases first and then decreases, exhibiting an optimal concentration range. This phenomenon stems from the balance of Coulomb interactions between ions: at low concentrations, repulsive forces between ions dominate, leading to higher mobility; in electrolytes with high salt concentration, the static dielectric constant of the electrolyte tends to decrease, and electrostatic interactions between ions increase with increasing salt concentration [219], which promotes the formation of an ion cluster. For instance, in aqueous electrolytes with high salt concentration, anions and cations form an ionic atmosphere composed of a central ion and outer layers of oppositely charged ions, hence decreasing the diffusion coefficient. Furthermore, such ion clusters may also block ion transport pathways, inhibiting the thermodiffusion effect of ions. Designing electrolyte structures with a gradient concentration distribution or introducing mixed ion systems containing multiple ions may be effective ways to suppress the formation of ion clusters [220].

For ionic thermogalvanic systems with redox-active ions, the ion concentration of redox couples can also directly adjust the thermopower. According to the Nernst equation, the thermopower ${S}_{{\mathrm{TG}}}$ of a redox couple can be expressed as Equation (2). The study by Nandal *et al*. [221] also mentioned that the thermopower of redox couples can be regulated from two aspects. One is to regulate the interaction parameters of redox couples in solvents, and the other is to regulate the concentration ratio of redox couples.

#### Controlling nanoconfinement effect

Nanoconfinement ion transport is a crucial process for achieving energy and material transfer in nature, which enables selective, efficient, and directional transport of specific ions, departing from the continuity assumption of classical ion transport theory [222]. The selective regulation of ion and atom transport through nanoconfinement effect has been extensively studied in research fields such as batteries [223–225] and environmental remediation [226–228]. Chen *et al*. [225] constructed two-dimensional nanochannels via the self-assembly of GO and P(VDF-TrFE-CTFE) on a lithium metal surface. These nanochannels selectively sieve solvent molecules through nanoconfinement and selective transport functions, thereby promoting Li⁺ desolvation and enhancing lithium salt dissociation. Furthermore, they facilitate Li⁺ transfer and regulate uniform Li deposition. This innovative interfacial architecture demonstrates exceptional mechanical stability and remarkable cycling performance, maintaining stable operation for over 2000 h at a current density of 2 mA cm^−2^. Xing *et al*. [226] developed a nanoconfinement catalyst derived from municipal sludge. They achieved *in situ* encapsulation of atomically dispersed iron centers within micro-mesoporous carbon substrates by employing selective hydrofluoric acid etching. This approach effectively prevents metal aggregation and modulates the local coordination structure of the active metal, shifting from Fe–N_4_ to Fe–N_3_O, leading to a significant enhancement in the efficiency and stability of reactive oxygen, as well as an improved pollutant degradation rate. In i-TE materials, the transport behavior of ions is regulated through nanoscale structures, thereby optimizing their thermodiffusion and thermopower. The core of this strategy lies in utilizing constructed nanostructures, such as directional nanopores, to spatially constrain the movement of ions, thus inhibiting disordered diffusion and selectively enhancing the directed migration of specific ions. For instance, Li *et al*. [119] achieved a thermopower of up to 24 mV K^−1^ for Na^+^ in natural nanoporous polymer cellulose i-TE gel. The confinement effect can significantly enhance the thermodiffusion rate of ions, as nanopores provide more regular transport channels, reducing the random movement of ions within the matrix.

Furthermore, nanoconfinement can enhance the interaction between Na^+^ and hydroxyl groups on the polymer matrix through interfacial effects, inducing the ordered arrangement of ions and thereby improving conductivity. Qian *et al*. [122] studied the thermopower of confined liquid electrolytes in closed systems and open systems connected to electrolyte reservoirs by solving the linear perturbation solution of the PNP equation. However, this classical PNP framework neglects critical non-classical effects—including ion–polymer synergistic dynamics, hydration entropy fluctuations, and non-equilibrium surface charge—that often dominate complex i-TE materials. While their numerical results align well across various EDL potentials and channel geometries, the study indicates that for PEs with significant diffusivity mismatches, confinement-induced thermopower enhancement is restricted to closed systems oriented perpendicular to temperature gradients. Considering the nanoconfinement effect and the influence of short-range electrostatic forces, Zhang *et al*. [229] revised the Nernst–Planck equation by incorporating the influence of steric effects, taking into account the ion distribution with Fermi concentration under nanoconfinement, described the EDL potential using the Poisson–Fermi equation, and theoretically and numerically simulated the significant enhancement of ion thermopower in nanoconfinement electrolytes by regulating the ion volume fraction, channel size, and channel surface potential, revealing the potential of nanoconfinement to enhance thermopower.

Researchers have recently proposed various methods to regulate the nanoconfinement effect. For example, by employing directional freezing [230] and directional stretching [231], as well as micellar construction, fast channels are formed in i-TE gels to selectively enhance the thermodiffusion of specific ions, thereby achieving an improvement in thermopower. Li *et al*. [84] added amphiphilic anionic surfactant sodium dodecylbenzenesulfonate (Na:DBS) to agarose i-TE gel. Due to the micellization effect of the amphiphilic anion DBS^–^, the gel matrix formed a unique porous structure. The hydrophilic benzenesulfonic acid group of DBS^–^ is attached to the hydrous agarose gel chains, while the hydrophobic alkyl chain points toward the center of the pores, forming tightly distributed micellar spheres. This reconstructed the transport channels of Na^+^ in the interstices of the polymer chains, achieving a thermopower as high as 41.8 mV K^−1^.

It is noteworthy that the ion size effect, concentration effect, and nanoconfinement effect are often interrelated. For example, small-sized ions with low molar concentration may exhibit more efficient migration behavior in nanoconfinement structures. Still, their low concentration and low thermodiffusion entropy can also affect the thermopower. Therefore, designing multilevel confinement structures that combine nanoscale pores of various sizes, or gradient confinement structures ranging from loose to dense, may be an effective strategy to further enhance ion transport selectively, thereby improving the thermopower and conductivity. Additionally, the study of ion transport mechanisms in i-TE devices still faces many challenges. First, accurately characterizing the kinetic behavior of ions, such as ion distributions, migration paths, and thermodiffusion coefficients in the i-TE gels with complex structures, remains a puzzle. Second, existing ion transport theoretical models, such as the Grotthuss mechanism, may have limitations in explaining the nanoconfinement effect, necessitating verification through advanced characterization methods. Moreover, the stability of ion transport performance, such as concentration polarization and electrode passivation, during long-term operation is also a significant factor limiting the development of i-TE devices. In the future, combining simulations with experimental research to develop i-TE systems with adaptive confinement effects will be a key direction to overcome this bottleneck, enhancing ion transport. The optimization of transport behavior of ions is a key task in improving the performance of i-TE materials. By deeply understanding the synergistic effects of ion size, concentration, and nanoconfinement and developing corresponding regulation strategies, it is expected to lay a solid foundation for the next generation of i-TE materials with high thermopower, high energy density, and high stability.

### Synergy between i-TE effects

Beyond the basic TD, TG, and TEx processes, the development of synergistic working mechanisms is essential for pushing the performance boundaries of i-TEs. Conventional i-TE devices are often restricted by their reliance on a single dominant mechanism, resulting in reduced thermopower, voltage stability, and adaptability. To overcome these bottlenecks, recent research has shifted toward coupling multiple driving forces, exploiting non-equilibrium processes, and integrating novel material platforms.

**Figure 10.**
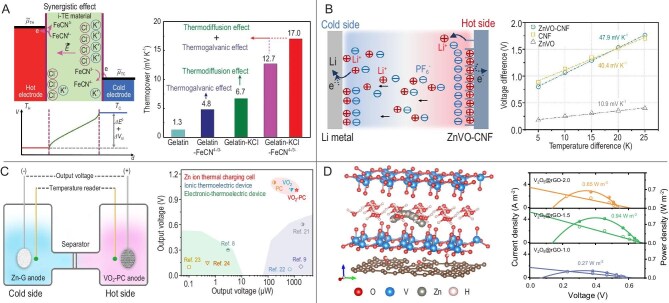
Representative advanced working modes of i-TEs. (A) Synergistic effect of thermodiffusion and redox reactions using a redox couple. Reproduced with permission from Ref. [4]. Copyright © 2020 The Author(s), exclusive licensee AAAS. (B) ZnVO-PC-based thermodiffusion and thermoextraction effects. Reproduced with permission from Ref. [234]. Copyright © 2022 Wiley-VCH GmbH. (C) VO-PC-based synergistic effect of thermodiffusion and thermoextraction. Reproduced from Ref. [47] under CC BY 4.0 license. Copyright © 2022 The Author(s). (D) Zn-based thermally chargeable system using a V_2_O_5_@rGO electrode. Reproduced from Ref. [237] under CC BY 4.0 license. Copyright © 2023 The Author(s).

One representative strategy is the hybrid operation mode, in which thermodiffusion-driven ion redistribution is coupled with temperature-dependent redox reactions. This strategy enables a synergistic enhancement of thermopower and output power, thereby achieving higher conversion efficiency compared with single-mechanism systems. For instance, in 2020, Han *et al*. [4] reported a quasi-solid-state i-TE material with an ultrahigh thermopower of 17.0 mV K^−1^ (Fig. 10A), realized by coupling the thermodiffusion of KCl with the negative temperature coefficient of the [Fe(CN)_6_]^4−/3−^ redox couple. The general design principle is to pair a p-type redox system (*α*< 0, ${S}_{{\mathrm{TG}}}$>0) and a p-type thermodiffusion system (${S}_{{\mathrm{TD}}}$>0), thereby maximizing the differential thermal voltage.

Another important direction is the thermoextraction effect, in which devices operate in a manner conceptually analogous to rechargeable batteries. Benefiting from advances in electrochemical energy storage, thermoextraction cells (TExCs) have recently garnered increasing attention [232]. Kobayashi *et al*. [233] first proposed a battery-type thermocell using paste-type electrodes, in which the thermoelectric effect was found to originate from the intercalation/deintercalation of Na^+^ ions. Extending this concept, Xu *et al*. [234] developed lithium–ion TExCs (LTExCs) based on graphite electrodes, where thermal charging drives the extraction of pre-intercalated Li^+^ from graphite and deposition onto lithium metal (Fig. 10B). Upon discharging, Li^+^ ions return to graphite layers, thereby enabling continuous low-grade heat harvesting. To enhance thermal kinetics, fibrous ZnVO-CNF composites with porous nanofiber networks have been introduced, offering rapid thermal intercalation/deintercalation and improved storage capacity.

Beyond monovalent ion systems, multivalent ions such as Zn^2+^, Mg^2+^, and Ca^2+^ provide denser charge storage. Among them, zinc metal is particularly attractive due to its intrinsic safety, high volumetric capacity, and low cost (∼65 USD kWh^−1^), making zinc–ion batteries one of the most promising candidates for large-scale applications [235,236]. Building on the robust electrochemical stability of zinc in aqueous electrolytes, Li *et al*. [47] recently introduced zinc–ion thermoelectrochemical devices that enable simultaneous low-grade heat harvesting and energy storage (Fig. 10C). A representative VO_2_-PC based device exhibits a thermopower of ∼12.5 mV K^−1^, a thermal power output of 1.2 mW, and an energy conversion efficiency of 0.95% under a 45 K temperature gradient. Meanwhile, Li *et al*. [237] further proposed a structural engineering strategy for electrode optimization, where the microstructure of hydrated vanadium pentoxide (VOMF) was effectively tailored using surfactants (Fig. 10D). As a result, the VOMF-based device achieved a thermovoltage of ∼0.6 V, a high thermopower of 6.4 mV K^−1^, and an areal power density of up to 1.8 W m⁻^2^ under a 40 K temperature gradient by enhancing ion diffusion and charge transfer. These findings demonstrate that rational structural regulation of electrode materials, coupled with the inherent advantages of multivalent-ion chemistry, provides an effective pathway to push the performance boundaries of thermoelectrochemical devices.

### Swelling and pressure effect

The performance of i-TE materials and devices is profoundly influenced by hydration-induced swelling and externally applied pressure. These factors modulate ion transport, interfacial properties, and ultimately energy conversion efficiency. A comprehensive understanding of these effects is essential for designing durable and high-performance i-TE systems.

#### The impact of water on TE performance

Water plays a dual role in i-TE materials, particularly in hydrogel-based systems. On one hand, hydration is crucial for facilitating ion dissociation and mobility. The presence of water molecules solvates ions, reduces energy barriers for ion transport, and enhances ionic conductivity. Hydrogels with high water content often exhibit superior thermopower due to the pronounced thermodiffusion of ions under a temperature gradient. For example, Zeng *et al*. [238] showed that the strong hydration of LiCl enhances the interaction between PAAm hydrogel and water, generating a high ionic thermopower of 11.3 mV K^–1^ and a maximum power density of 167.90 mW m^–2^ under a temperature difference of 20 K. This is attributed to the high entropy difference between cations and anions in aqueous environments, which amplifies the intrinsic ionic thermopower.

On the other hand, excessive water uptake can lead to significant swelling, which may adversely affect the material’s mechanical integrity and dimensional stability. Swelling can increase the inter-ionic distances, potentially diluting ion concentration and reducing effective ionic conductivity. Li *et al*. [239] have presented a multi-effect-coupling thermal-stimulus (MECtherm) model to consider the effects of multi-phases and multi-physics on the volume phase transition of ionic thermo-sensitive hydrogels in response to thermal stimulus. Moreover, uncontrolled swelling introduces inhomogeneities in the material, leading to inconsistent thermoelectric properties and poor device reproducibility. Therefore, optimizing the cross-linking density and polymer network structure to balance water retention and dimensional stability is a key strategy in material design. For instance, the hydrophobic ionogels incorporating methyl methacrylate (MMA) and ILs such as [BMIM][PF_6_] have demonstrated remarkably low swelling rates (as low as 2.8%) while maintaining high ionic thermopowers and power densities, enabling stable underwater operation [240]. Notably, the impact of the water concentration gradient between the hot and cold ends on TE performance cannot be overlooked, particularly for highly hygroscopic materials (Fig. 10D).

#### Osmotic pressure-driven ion diffusion

Osmotic effects, arising from concentration gradients of ionic species, are a major driving force for ion diffusion in i-TE systems. Under a temperature gradient, the non-uniform distribution of mobile ions generates not only a thermal potential but also an osmotic pressure difference. This osmotic pressure can significantly enhance ion migration, particularly in porous or gel-type electrolytes where the matrix allows for solvent flow [241,242].

The coupling between thermal diffusion and osmotic flow offers a promising route to enhance the overall energy conversion performance. Similar to the thermal-osmotic ionogel (TOI) proposed by Campbell *et al*., waste heat is used here to drive the phase transition of flexible ionic crystals to generate an ion gradient, followed by selective ion diffusion [243]. For instance, in certain hydrogel i-TE devices, the TD effect and osmotic pumping can work synergistically to achieve higher current outputs and improved power generation. The gel-state TOI can achieve much higher thermo-diffusive thermopower because the gel-state TOI composite has dense hydrophilic groups (such as amide groups in PAM polymer) and can form intermolecular hydrogen bonding with anions and hinder their diffusion to create a cation concentration gradient [87,244]. Theoretical models based on Onsager’s reciprocal relations have been developed to describe the coupled transport of heat, charge, and mass, highlighting the role of osmotic pressure in enhancing the effective thermopower and ionic conductivity. Managing these osmotic dynamics through material selection and structural control is critical for maximizing i-TE efficiency. The concept of ‘osmotic heat engines’ further underscores the potential of harnessing osmotic pressure for low-grade thermal energy harvesting.

#### Pressurized electrolyte–electrode interface

The interface between the electrolyte and the electrode is a critical zone where pressure, either externally applied or internally generated, can dramatically influence charge transfer and overall device performance. Applying external pressure can enhance interfacial contact, reduce electrical contact resistance, and facilitate more efficient ion adsorption/desorption at the electrode surface. Ho *et al*. proposed a self-healing i-TE device using zwitterionic (ZI) copolymer-based n/p-type i-TE gels integrated by liquid-metal based pressure-driven selective active electrode [245]. With the pressure-induced ion–polymer interactions, the device will be easily self-healed from minor damage. This is particularly important in flexible or wearable applications where device deformation may lead to unreliable contacts.

Conversely, internal pressure from swelling may cause delamination, increased interfacial resistance, or even mechanical failure at the electrode–electrolyte junction, which is a common concern in flexible electrochemical devices. In electrochemical capacitors or i-TE generators employing porous electrodes, swelling-induced pore blockage can hinder ion accessibility, reducing active surface areas and impairing performance. Recent studies have shown that pre-pressurization or the use of compliant electrode materials can mitigate these adverse effects, as seen in self-healable electrodes composed of liquid metal and polymers, which have been demonstrated to maintain interfacial integrity under cyclic mechanical stress [245]. Furthermore, understanding the role of pressure in modulating the EDL structure and dynamics is vital, as changes in local pressure can alter the EDL capacitance and ion mobility at the interface. All-atom simulations have revealed that mechanical deformation of electrodes (for example, compression, bending) significantly redistributes the local electric field and ion arrangement within the EDL [246].

Notably, innovative systems such as piezoelectric-augmented ionogels demonstrate that applied pressure can not only improve contact but also actively enhance i-TE performance [247]. In such systems, the piezoelectric field generated under pressure works synergistically with the thermal gradient to further promote ion separation, leading to a significant increase in thermopower (for example, from −4.4 to −6.9 mV K^−1^ for n-type systems). This interplay between mechanical pressure and electrochemical response highlights the potential of multi-field coupling in next-generation i-TE devices. Additionally, self-healable electrodes composed of liquid metal, waterborne polyurethane, and PVA have been developed to maintain interfacial integrity under cyclic pressure, enabling fully healable TEGs with stable output.

## CRITICAL APPLICATIONS

### Power generation using i-TE

#### Power generation for wearable sensors

**Figure 11.**
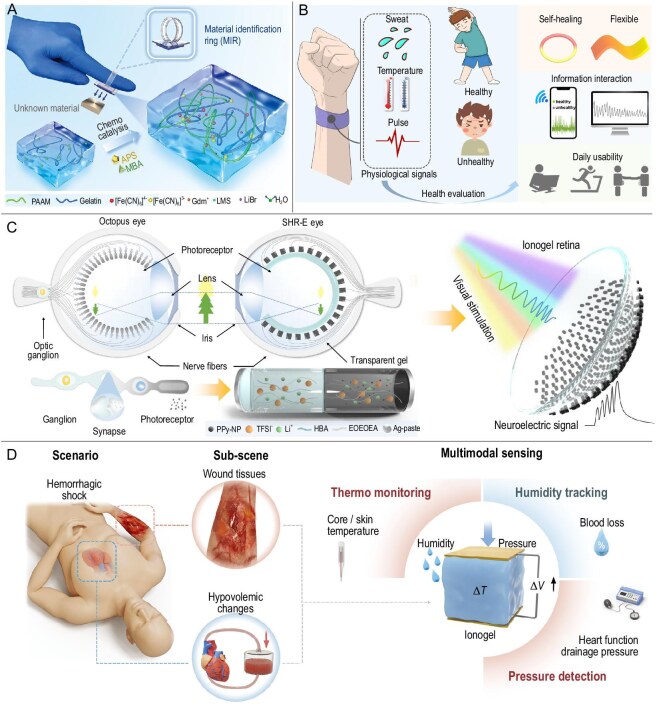
Various power generation applications using i-TE. (A) Self-powered machine-learning-assisted material identification enabled by a thermogalvanic dual-network hydrogel with a high thermopower. Reproduced with permission from Ref. [248]. Copyright © 2024 Wiley-VCH GmbH. (B) Thermoelectric hydrogel electronic skin for passive multimodal physiological perception. Reproduced with permission from Ref. [249]. Copyright © 2024 ACS. (C) A bionic self-driven retinomorphic eye with ionogel photosynaptic retina. Reproduced with permission from Ref. [250] under CC BY 4.0 license. Copyright © 2024 The Author(s). (D) Piezoelectric-augmented thermoelectric ionogels for self-powered multimodal medical sensors. Reproduced with permission from Ref. [247]. Copyright © 2024 Wiley-VCH GmbH.

The flexible stretchable i-TE gels have the unique advantage for self-powered wearable sensors, which directly convert low-grade thermal energy from human body or environment into electricity. In 2016, Prof. Jun Zhou pioneered the first demonstration of a flexible gel-based i-TE device [182]. Recently, Li *et al*. developed a thermogalvanic dual network hydrogel (TG-DNH) system that achieves an exceptionally high thermopower of 4.01 mV K^−1^ using a [Fe(CN)_6_]^4−/3−^ redox couple and optimized electrolyte formulation (Fig. 11A) [248]. One key advantage of this system is its rapid response to temperature variations. When integrated into a wearable ring, it generates sufficient operating voltage by harnessing the temperature difference between the skin and environment. Experimental results demonstrate the device delivers a maximum power density of 0.99 W m^−2^ with an instantaneous power output of 51.5 μW under a 25 K temperature gradient. Moreover, the system retains a high output current after 800 bending cycles, exhibiting excellent durability. The TG-DNH-based self-powered material recognition system, when combined with machine learning (ML), enables highly efficient and precise material identification. This technology shows significant potential for future applications in human–machine interaction (HMI) and artificial intelligence, providing innovative solutions for wearable haptic sensing.

For synergistic integration of energy harvesting, signal sensing, and data processing, Tian *et al*. developed a flexible hydrogel wristband sensor (Fig. 11B) [249]. By incorporating a [Fe(CN)_6_]^4−/3−^ redox pair and glycerol-LiCl binary system, the device achieves a high thermoelectric performance (2.04 mV K^−1^), 91% self-healing efficiency within 10 min, and a conductivity of 2.56 S m^−1^. During body temperature monitoring, it produces an open-circuit voltage of 8.23 mV and a short-circuit current of 114.2 μA at a 5 K temperature difference, with signal variations sensitively detecting abnormal temperature fluctuations. For pulse monitoring, the hydrogel wristband stably records radial artery waveforms, resolving heart rate characteristics (60 bpm) and biphasic pulse structures. Simulated sweat tests reveal concentration-dependent responses to electrolytes (Na^+^, K^+^, and Ca^2+^), with Ca^2+^ triggering a distinct transition from transparent to opaque. The system achieves synchronous multi-parameter monitoring by decoupling temperature (voltage), pulse (high-frequency current), and sweat (stepwise current) signals. The wireless transmission of physiological data to smart devices validates the Internet of Things (IoT) healthcare potential. With anti-freezing (−32.79°C operational flexibility), anti-desiccation (70% water retention after 10 days), and self-healing properties, the device maintains functionality in extreme conditions, enhancing wearable reliability. This integrated platform combines thermoelectric conversion, physiological sensing, and energy autonomy, enabling real-time self-powered multimodal monitoring—a paradigm shift in unified wearable systems.

These groundbreaking advances collectively drive the rapid development of thermoelectric technology in wearables, marking a transformative leap toward sustainable, self-powered wearable ecosystems. From fundamental material breakthroughs, such as the development of high thermopower gels, to sophisticated device optimization strategies, i-TE systems are overcoming long-standing energy challenges in wearable electronics. The integration of advanced ML algorithms further enhances their functionality, enabling real-time signal processing, pattern recognition, and adaptive responses, which are critical for applications in personalized healthcare, smart prosthetics, and human–machine interfaces. In the realm of remote healthcare monitoring, i-TE-powered wearables offer continuous, non-invasive physiological tracking without reliance on external power sources.

#### Power generation for embedded sensors

I-TE materials are revolutionizing embedded medical devices through their distinctive energy conversion mechanisms, enabling novel approaches for stable long-term *in vivo* monitoring. These materials address the persistent challenge of battery replacement in conventional embedded sensors while achieving tissue-device symbiosis via advanced biocompatible engineering.

Li *et al*. harnessed the i-TE effect in a PPy-gel/pure-gel heterojunction structure to create temperature gradients under light illumination [250]. This design drives selective migration of Li^+^ and TFSI^−^ ions, enabling self-powered sensing without external energy input (Fig. 11C). The device achieves a remarkable thermopower of 1.57 mV K^−1^ and maintains a response current of 75.87 μA W^−1^ under weak illumination (2.08 μW mm^−2^), ensuring reliable performance in challenging conditions. As an embedded sensor, the system exhibits exceptional biocompatibility and surgical adaptability. Its flexible ionogel substrate, with an elastic modulus matching biological tissues, conforms seamlessly to retinal curvature. The modular 5-pixel array allows plug-and-play localized repairs, with post-embedding photocurrent recovering to healthy levels. Notably, the device emulates neural functions through synaptic plasticity (paired-pulse facilitation index, 153%) and dynamic memory characteristics (relaxation time, 64.36 s). These features enable both static imaging and motion trajectory tracking, resolution of 3.4 cm min^−1^. By eliminating dependence on external power sources and leveraging the inherent biosafety of ionic conductors, this self-powered embedded system overcomes limitations for long-term deployment, paving the way for advanced smart prosthetics, neural interfaces, and adaptive machine vision technologies.

To advance from single-signal detection to multimodal physiological parameter monitoring, Pai *et al*. proposed an ionogel sensor with exceptional integration capabilities (Fig. 11D) [247]. By embedding the sensor in silicone catheters or vacuum drainage sponges, the system enables three-dimensional synchronous monitoring of wound exudate pressure (5.3–24.0 kPa range), local temperature (30–40°C), and humidity (0.033 mV K^−1^%^−1^). Notably, in a porcine hemorrhagic shock model, the sensor array embedded in the jugular vein tracked real-time changes in pulmonary artery wedge pressure. The high sensitivity (0.28 mV kPa^−1^) not only meets clinical standards for central venous pressure monitoring but also enables continuous dynamic monitoring of intravascular pressure. This intravascular-embedded design overcomes limitations of external monitoring by capturing subtle cardiac cycle-induced pressure fluctuations. The study further revealed that the blood environment enhances the sensor’s thermoelectric performance (10-fold increase to −1.96 mV K^−1^), attributed to the promotion of ion migration by blood electrolytes and the sustained thermal gradient provided by the stable temperature field in blood vessels. This property ensures stable self-powered operation during prolonged intravascular use, offering an innovative platform for cardiovascular monitoring and shock resuscitation.

From superficial light sensing in bionic eyes to intravascular multimodal physiological signal acquisition, thermoelectric embedded sensors are progressively overcoming challenges in biocompatibility, environmental interference, and signal stability. These advancements not only resolve the bottleneck of traditional embedded sensors’ dependence on external power sources but also enable functional integration and performance optimization through biomimetic designs and intelligent algorithms. The potential applications will extend beyond the medical field, enabling real-time tactile feedback for bionic prosthetics in soft robotics and tracking pollutant exposure in environmental monitoring. With ongoing research in miniaturization, intelligence, and multifunctionality, these self-powered systems are poised to become the core of next-generation medical devices. By delivering reliable, long-term, and minimally invasive monitoring capabilities, they will drive the development of personalized medicine and ultimately catalyze fully autonomous bioelectronic therapies, fundamentally transforming disease diagnosis, treatment, and health management paradigms in the 21st century.

### Cooling and refrigeration using i-TE

#### Active cooling

**Figure 12.**
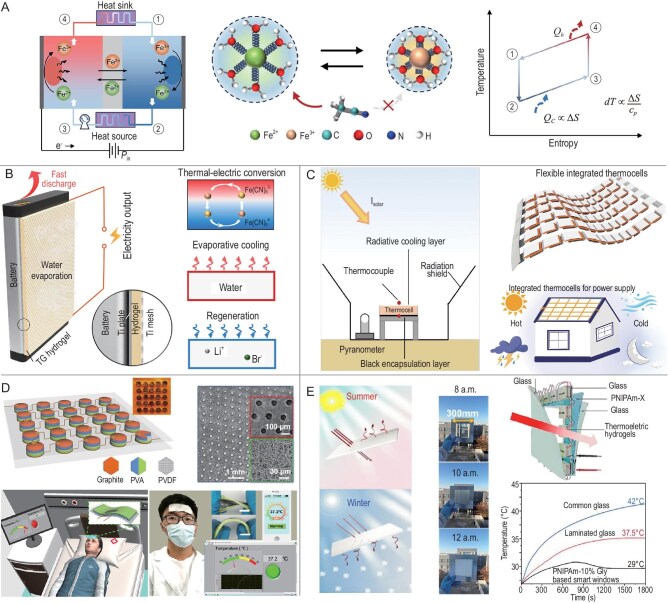
Various cooling and refrigeration applications using i-TEs. (A) Schematics of a thermogalvanic system using Fe^2+^/Fe^3+^ electrolyte for electrochemical refrigeration and the solvation structure of Fe^2+^ and Fe^3+^ with the selective interaction of MeCN, and the corresponding temperature-entropy diagram. Reproduced with permission from Ref. [153]. Copyright © 2025 Elsevier Inc. (B) Schematic of the experimental setup and schematic of the working principle of the smart thermogalvanic hydrogel. Reproduced with permission from Ref. [258]. Copyright © 2020 ACS. (C) Scheme of the setup for testing performance under sunlight, and flexible integrated thermocells for collecting thermal energy under different environmental conditions. Reproduced with permission from Ref. [259]. Copyright © 2024 Wiley-VCH GmbH. (D) Schematic of device structure, schematic diagrams, physical application scenarios, and human–machine interface of the integrated cooling system. Reproduced with permission from Ref. [260]. Copyright © 2021 ACS. (E) Schematic of working principle and device architecture of smart windows, physical demonstrations, and comparative cooling performance with other materials. Reproduced with permission from Ref. [261]. Copyright © 2024 ACS.

Different from e-TE cooling [251], i-TE refrigeration is realized by utilizing electricity, exploiting the reversible, electrically induced entropy change effect in redox couples [8,66,252]. The underlying mechanism is the large entropy change accompanying redox reactions of couples such as Fe^2+/3+^ at the electrode interface, which drives simultaneous endothermic and exothermic processes. Zeng *et al*. [153] proposed a synergistic solvent and anion engineering strategy to enable high-efficiency electrochemical refrigeration by reconstructing the solvation structures of iron ions (Fe^2+/3+^). The researchers employed perchlorate ions (ClO_4_^–^) as weakly coordinating anions to weaken iron ion–anion interactions, while introducing nitrile solvents (such as MeCN) to selectively modulate the solvation shell of Fe^2+^ (Fig. 12A). Experimental results demonstrated that this design increased the temperature coefficient from a baseline of 1.73 to 3.73 mV K^−1^. This asymmetric reconstruction enlarges the entropy difference between solvation structures of Fe^2+^ and Fe^3+^, thereby significantly enhancing the entropy change of the redox reaction.

In addition to the temperature coefficient, the specific heat capacity of the electrolyte is another critical parameter constraining refrigeration efficiency. Traditional aqueous electrolytes show a sluggish cooling response due to water’s high specific heat capacity. Zeng *et al*. [153] innovatively employed a MeCN/H_2_O binary solvent system, leveraging the low specific heat capacity of MeCN to reduce the average specific heat capacity of the electrolyte. Notably, its electrolyte-specific coefficient of performance (COP) reaches 14.3 at low voltages, significantly surpassing that of conventional semiconductor thermoelectric refrigeration systems and highlighting its potential for integration with micro-energy technologies [253–255]. This work overcomes key bottlenecks in thermogalvanic refrigeration through solvation entropy engineering. The record-high temperature coefficient, synergistically coupled with substantially reduced specific heat capacity, elevates refrigeration power by 70% and achieves a 1.42 K temperature drop. The dual anion-solvent engineering strategy establishes an effective guiding principle for next-generation i-TE design, promoting high efficiency of electrochemical refrigeration systems [256,257].

#### Passive cooling

Passive cooling utilizes natural thermodynamic processes to dissipate heat without external energy, driven by environmental temperature differentials that induce autonomous directional migration of heat carriers. Within i-TE systems, this technology simultaneously accomplishes waste heat dissipation and energy harvesting by directly converting temperature gradients into electrochemical energy. Water evaporation is a key passive cooling mechanism. However, dissipating heat from electronic devices requires frequent water replenishment, which can expose the sensitive electronics to unwanted moisture. The persistent demand for electronic device cooling and waste heat recovery is addressed by a polyacrylamide-based thermogalvanic hydrogel developed by Pu *et al*. This system decouples i-TE conversion from water phase cycling to achieve simultaneous cooling and power generation [258]. The gel incorporates a [Fe(CN)_6_]^4−/3−^ redox couple and hygroscopic LiBr (Fig. 12B). When applied to a 5000 mAh smartphone battery, a 2 mm thick hydrogel reduces temperature by 20°C at a 2.2 C discharge rate. The evaporation process absorbs approximately 245 J g^−1^ heat for efficient thermal removal, while the temperature difference simultaneously drives redox reactions to produce 14.5 mV voltage and 1.4 mA current, recovering 5 μW electrical power. Post operation, the gel regenerates hygroscopically within 193 min, with harvested energy charging capacitors to 13.5 mV.

Conventional i-TE devices are constrained by the challenge of maintaining continuous temperature differences and their narrow operational temperature range. Yang *et al*. [259] proposed a self-sustaining temperature difference strategy based on passive radiative cooling. By incorporating a high thermopower thermogalvanic ionogel, they achieved power generation across an ultrawide –40–90°C temperature range. The device features a sandwich architecture: the top layer is a hierarchically porous P(VDF-HFP); the bottom layer is a carbon nanotube-modified bifunctional encapsulation layer; and the middle layer comprises the THG-ionogel electrolyte (Fig. 12C). Critically, it maintains continuous electricity generation across diverse weather conditions—including rain, snow, and variable cloud cover—overcoming the traditional dependence on external heat sources for thermoelectric devices. Through material innovation and structural design, these two different technologies can achieve a deep integration of passive thermal management and thermoelectric conversion, indicating that passive management is not merely a cooling strategy but, through thermodynamic engineering, a crucial driver for energy harvesting.

#### Flexible electronics and intelligent thermal regulation

Intelligent thermal regulation represents an advanced frontier in i-TE technology, aiming to dynamically manage heat transfer while simultaneously converting waste thermal energy into electricity. This dual functionality is particularly compelling for applications requiring autonomous operation and adaptive responses to varying thermal loads, such as in wearable electronics and smart building systems. In thermal management applications, a key focus is leveraging the human body as both a thermal source and a management target. The inherent temperature gradient between the skin and ambient environment provides a sustainable energy source for self-powered devices. Bai *et al*. [260] developed a PVA hydrogel thermoelectric device using a Fe^2+/3+^ redox couple. By integrating a porous PVDF separator as a thermal barrier, the thermopower increased by 36% to 0.79 mV K^−1^. The forehead-mounted patch enables real-time temperature monitoring with >60 μA current variation and 0.1 K detection limit, while simultaneously reducing inflammation site temperatures through high specific heat capacity. The device maintained stable current output after 3 days of dehydration at 50% humidity, overcoming traditional semiconductors’ rigidity and cost limitations (Fig. 12D).

Beyond personal wearables, i-TE materials are being integrated into architectural elements for energy-efficient climate control. Smart windows equipped with thermo-responsive hydrogels, such as those based on PNIPAM, offer passive temperature regulation. Xie *et al*. [261] achieved precise regulation of coloration transition temperature (24–43°C) in smart windows by modulating the polymer network with N,N-dimethylacrylamide (DMAA) and glycerol. The smart windows innovatively integrate i-TE hydrogel modules that generate electricity from glass surface waste heat (Fig. 12E). Experimental testing shows that a 300 × 300 mm window outputs 1.25 V under noon temperature differences, powering a 0.15 W bulb, achieving dual objectives of thermal energy recovery and extended device lifespan. These developments underscore a strategic shift from mere thermal regulation to integrated systems that perform sensing, management, and energy harvesting concurrently. By embedding i-TE functionality directly into flexible substrates and building materials, this approach lays the foundation for autonomous, energy-aware applications that contribute to both personal thermal comfort and large-scale energy sustainability.

### Multi-functional sensing using i-TE

#### Temperature sensor

I-TE effect not only provide electricity, but also could be used as the temperature sensing. When integrated with ML algorithms, thermoelectric sensors enable sophisticated signal analysis and target recognition, further expanding their potential applications in innovative healthcare, the IoT, and artificial intelligence [284,285]. These studies establish a critical foundation for developing high-performance, multifunctional self-powered wearable sensors.

Temperature monitoring is vital in healthcare, environmental science, and industry for disease prevention, energy efficiency, and safety. While hydrogel-based flexible temperature sensors utilizing resistive [262,263], capacitive [264], and thermochromic sensing mechanisms [265] have been widely studied, their electrical outputs are often influenced by strain, humidity, and gases, leading to multi-signal interference. In contrast, i-TE materials generate voltage through ionic thermodiffusion or thermogalvanic reactions, producing a single output that minimizes environmental noise. This makes i-TE materials a promising direction for accurate and robust flexible temperature sensing.

**Figure 13.**
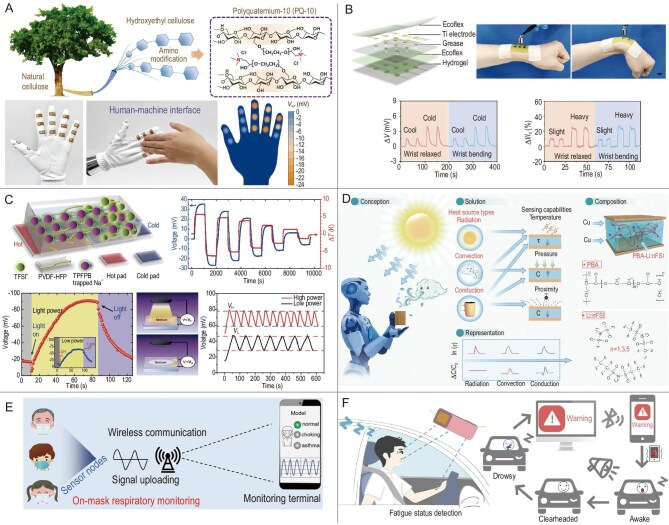
Various applications of i-TEs as multi-functional sensors. (A) Schematic of the temperature recognition application using the ultrasensitive flexible thermal sensor array based on high-thermopower i-TE hydrogel. Reproduced with permission from Ref. [135] under CC BY 4.0 license. Copyright © 2023 The Authors. Published by Wiley-VCH GmbH. (B) Dual-stimuli-responsive thermoelectric hydrogel sensing array for temperature and pressure monitoring. Reproduced with permission from Ref. [266]. Copyright © 2022 ACS. (C) Schematic diagram of p–n type conversion principle based on i-TE materials and its application as a heat source sensor. Reproduced with permission from Ref. [137] under CC BY 4.0 license. Copyright © 2022 The Author(s). (D) Schematic of an i-TE-based multi-sensing source identification sensor capable of simultaneous temperature, proximity, and pressure detection. Reproduced with permission from Ref. [267] under CC BY-NC-ND 4.0 license. Copyright © 2024 The Authors. Published by Elsevier Inc. on behalf of Youth Innovation Co., Ltd. (E) Schematic of the self-powered breathing monitoring mask based on an adaptive dual-network thermoelectric hydrogel for stable operation under extreme conditions. Reproduced with permission from Ref. [271]. Copyright © 2022 ACS. (F) Schematic of edible and controllably peelable hydrogels based on i-TE materials for multipotential monitoring to prevent fatigued driving. Reproduced with permission from Ref. [272]. Copyright © 2024 RSC.

Recent research has focused on enhancing the performance of i-TE materials through the development of new materials and innovative structural designs. For example, Han *et al*. [135] developed a PQ-10/NaOH hydrogel achieving a record p-type thermopower of 24.17 mV K^−1^ in biopolymer systems. Its quaternary ammonium groups suppress anion diffusion, while Na^+^ ion migration under a thermal gradient generates a proportional voltage. The device demonstrates remarkable environmental stability, maintaining signal integrity for two months under ambient conditions. When integrated into flexible circuit boards, it forms a 5-pixel thermal array with a sensitivity of 2.7 mV K^−1^. The team extended this approach by fabricating a smart glove with fourteen integrated sensing nodes distributed along the digits of a prosthetic hand, as shown in Fig. 13A. The smart glove generated spatially resolved voltage signatures upon contact with objects, accurately reconstructing the thermal profile of the contacted surfaces while successfully detecting both the temperature and precise location of touch.

Beyond single-parameter temperature detection, Yang *et al*. [266] developed a stretchable and self-powered temperature-pressure dual-mode sensor by incorporating a [Fe(CN)_6_]^4−/3−^ redox couple into a polyacrylamide hydrogel, as shown in Fig. 13B. This thermoelectric hydrogel shows a thermopower of −1.21 mV K^−1^ and a pressure sensitivity of 0.056 kPa^−1^. The multifunctional sensor achieves precise recording of both thermal information and tactile perception on human skin by integrating electrode arrays. This work establishes a conceptual framework and systematic design for applications in stretchable artificial skin, interactive wearable devices, and intelligent robotics. Together, these advances illustrate how i-TE materials are advancing sensing capabilities through sophisticated material design. The stretchable thermogalvanic hydrogel demonstrates successful integration of temperature and pressure sensing—building the groundwork for multifunctional perception systems in next-generation wearable and robotic platforms.

#### Heat source sensor

Heat serves as a fundamental mode of energy transfer that critically influences both natural and engineered environments. To effectively monitor and manage thermal distribution, heat source sensors have become indispensable instruments. Traditional inorganic thermoelectric sensors are limited by low thermopower, rigidity, and high cost. In contrast, i-TE materials have emerged as a promising alternative due to their high thermopower, flexibility, eco-compatibility, and low cost, showing great potential for heat source sensing applications.

The performance optimization and mechanism elucidation of i-TE materials are fundamental to their application in heat source sensors. The fabrication of the all-solid-state flexible polymer composite PVDF-HFP/NaTFSI/PC (PhNP) by Huang *et al*. represents an early breakthrough in this field. By regulating the transport of Na^+^ ions, they achieved a wide thermopower tuning range from +20 to –6 mV K^−1^ and observed the p–n conversion phenomenon in all-solid-state i-TE polymers for the first time [137], as shown in Fig. 13C. With just 4 p–n leg pairs, the device shows high sensitivity to light-generated heat. A non-contact thermal sensor with excellent response was also developed, proving the potential of i-TE materials for heat-sensing applications.

Beyond fundamental research on i-TE sensors, the heat source recognition sensor has also greatly advanced biomimetic applications. Liu *et al*. [267] developed a heat-source recognition sensor based on a poly(butyl acrylate)–lithium bis(n-fluoroalkylsulfonyl)imide (PBA-Li:nFSI, *n* = 1, 3, 5) ionic gel, which discriminates among radiative, convective, and conductive heating modes via ionic relaxation dynamics, as shown in Fig. 13D. Integrated with an e-skin capable of isothermal regulation, the sensor confers multidimensional thermal perception to robots, enabling them to identify both the type and temperature state of different heat sources—such as sunlight (radiative), a hairdryer (convective), or a handshake (conductive). This biomimetic system closely replicates the thermosensory function of human skin, offering a transformative approach for intelligent robotics and prosthetic applications.

#### Gas sensor

Gas monitoring plays a critical role in detecting physiological signals, with applications in medical diagnosis [268], health management [269], and safe protection [270]. In recent years, i-TE materials have emerged as a promising focus for gas monitoring research due to their unique thermoelectric conversion properties. Their high thermal sensitivity, low cost, and flexible properties enable the development of self-powered, wearable sensors for breath analysis.

Zhang *et al*. developed a double-network i-TE hydrogel utilizing a [Fe(CN)_6_]^4–/3–^ redox couple for self-powered respiration monitoring, as shown in Fig. 13E. The hydrogel exhibits excellent anti-freezing and anti-drying properties, ensuring stable operation under extreme conditions. Integrated into a face mask, it enables real-time, wireless monitoring of respiratory signals and can distinguish between normal breathing, choking, and asthma patterns. This self-powered system monitors both respiratory rate and depth while maintaining functionality at low temperatures, demonstrating strong environmental adaptability for wearable health monitoring [271].

Besides, Zhang *et al*. [272] further proposed an i-TE hydrogel based on Gelatin/Glycerol (Gel/GL) for self-powered multi-point fatigue monitoring, as shown in Fig. 13F. This hydrogel not only exhibits excellent thermoelectric properties but also possesses temperature-responsive adhesiveness. The attachment of hydrogel patches to positions such as the outer corners of the eye, the philtrum, and the artery enables the simultaneous monitoring of physiological signals, including respiration, blinking, yawning, and pulse [272]. Thereby, the high thermal sensitivity, low cost, and flexible properties of the i-TE materials demonstrate that they’re highly promising for monitoring respiratory gases.

### Innovative applications of i-TE

#### I-TE materials for future smart interaction

The rapid advancement of intelligent HMI technologies has made them a central focus in artificial intelligence and IoT research [273,274]. Within this domain, i-TE materials demonstrate significant application potential owing to their unique self-powered capability, high sensitivity, and exceptional mechanical flexibility. This intrinsic functionality allows continuous monitoring and interaction without external power sources. Recent research advances have substantiated the viability of i-TE materials across multiple fields, underscoring their promise in enabling more autonomous HMI systems [275–277].

**Figure 14.**
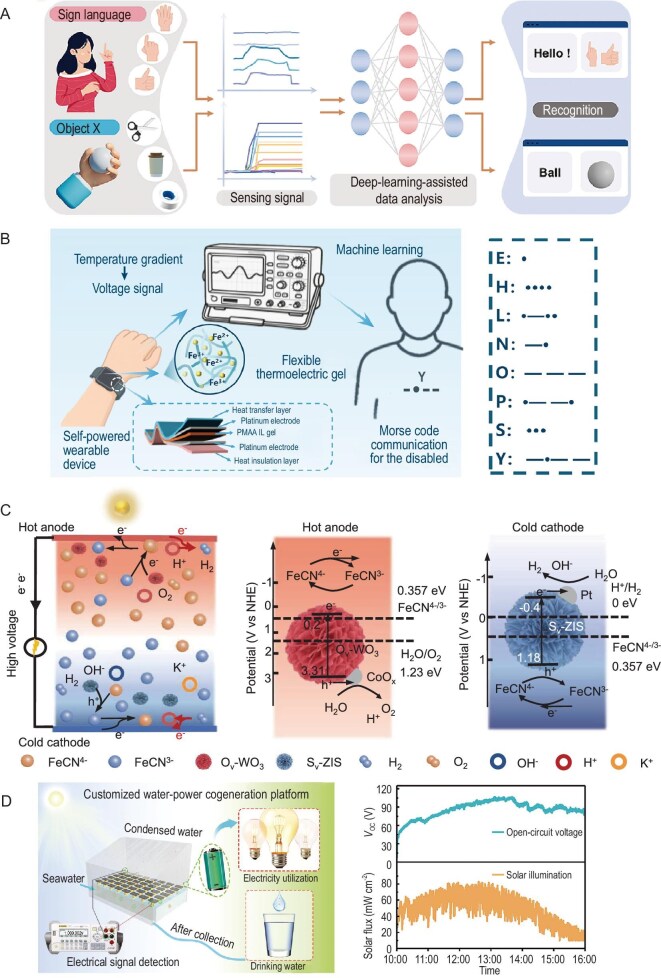
Representative innovative applications of i-TE devices. (A) Schematic diagram of sign language recognition interaction. Reproduced with permission from Ref. [278]. Copyright © 2024 Elsevier B.V. (B) Schematic illustration of the thermoelectric self-powered accessible communication system. Reproduced with permission from Ref. [279] under CC BY 4.0 license. Copyright © 2025 The Author(s). Published by Wiley-VCH GmbH. (C) Schematic of the working mechanism in photocatalytically enhanced i-TE device for simultaneous waste heat harvesting and generation of hydrogen. Reproduced with permission from Ref. [285]. Copyright © 2023 The Author(s), exclusive licensee AAAS. (D) The integration and applications of Bionic IEHVG. Reproduced with permission from Ref. [123]. Copyright © 2024 Wiley-VCH GmbH.

The human hand provides rich sensory information essential for environmental interaction. Signals generated by hands-on wearable equipment can be converted and processed to enable information transfer and sharing in HMI, healthcare monitoring, and motion tracking. As shown in Fig. 14A, Yang *et al*. [278] developed an autonomous gesture-recognition glove utilizing dynamically crystallized ion-injected thermoelectric gel, which synergistically integrates thermoelectric and piezoresistive sensing mechanisms. Miniaturized i-TE modules were integrated onto critical glove joints to enable multimodal signal acquisition, generating stable current signals with a sensitivity of 0.06 kPa^−1^ under concurrent thermal (${\mathrm{\Delta }}T$ = 1 K) and mechanical (1 kPa) stimulation. Deep learning algorithms allow the real-time classification of intricate gestures with 98.2% accuracy. This system pioneers dual-mode applications: it provides real-time sign language translation for hearing-impaired users while achieving sub-millimeter motion tracking through seamless VR/AR integration, thereby advancing naturalistic HMI.

Thermoelectric HMI technology demonstrates broad applicability across both communication systems and wrist-worn devices. To leverage this advantage, Kong *et al*. [279] developed a poly (methacrylic acid) (PMAA)-based IL gel using an innovative solvent exchange approach, yielding high ionic conductivity (13.45 S m^−1^) and a stable thermopower (−4.67 mV K^−1^). This gel was integrated into a self-powered Morse code platform that converts thermal gradients into electrical signals (Fig. 14B). ML algorithms were implemented to achieve high-accuracy decoding of Morse code, with the logistic regression model attaining 100% accuracy on independent test sets. Collectively, this integrated hardware-software framework establishes a novel solution for barrier-free communication, particularly demonstrating feasibility for applications requiring robust signal interpretation under variable conditions. These advances collectively illustrate how i-TE materials are moving from single-function sensors to core components of intelligent interactive systems [280,281]. By providing autonomous power and high-fidelity signal acquisition, they lay a practical foundation for next-generation HMI technologies that are more adaptive, accessible, and integrated into daily life and specialized environments alike.

#### Integrated water–electricity–hydrogen generation

Against the backdrop of global carbon neutrality imperatives, i-TE materials are shifting the paradigm of low-grade heat utilization from single-output processes toward integrated multi-output systems. This transformation is epitomized by the integrated water–electricity–hydrogen (IWEH) system, which orchestrates the synergistic coupling of three distinct energy-resource conversion processes: i-TE conversion, water electrolysis, and desalination via selective ion transport [282–284].

The operational principle of IWEH systems is based on the capacity of i-TE to harvest low-grade thermal energy through thermally driven ion migration. Confronting the persistent limitation of redox ion concentration gradient dissipation, Wang *et al*. [285] pioneered an *in situ* photocatalytic regeneration methodology (Fig. 14C). This innovation is implemented using a hybrid electrode that incorporates TiO_2_-based photocatalysts and interfaces with a Fe^2+/3+^ redox electrolyte, enabling continuous synergy between thermal, electrical, and chemical energy flows within a single device. As temperature gradients induce directional ion flux to generate initial voltage, incident photons simultaneously trigger water oxidation and reduction reactions through photogenerated electron–hole pairs, perpetually replenishing the decaying ion concentration gradient. Material-level characterization validates an exceptional thermopower of 8.2 mV K^−1^ concurrent with a solar-to-hydrogen conversion efficiency of 0.4%. This integrated device represents the first experimental realization of complete thermal-to-electrical-to-chemical energy cascading in a functional unit.

Bionic interfacial evaporation-driven hydrovoltaic generators (IEHVGs) achieve high resource conversion efficiency comparable to desalination units [286–288]. This enables the miniaturization and distributed deployment of IWEH systems through structural innovation. Inspired by natural transpiration, IEHVGs incorporate vertically aligned fluid channels and gradient hydrophobic evaporation interfaces to generate power and desalinate seawater simultaneously [123]. The working mechanism leverages water evaporation at functionalized interfaces (gradient hydrophobicity/high surface charge) to spontaneously establish localized thermal/concentration gradients. By coupling thermal diffusion with ion-selective transport, the system directly converts evaporation energy into electricity while simultaneously desalinating seawater (Fig. 14D). Experimental studies demonstrate that a single device delivers a power density of 45.6 μW cm^–2^ under simulated solar irradiation of 1 kW m^–2^. This closed-loop system processes seawater through evaporation and thermoelectric conversion to produce freshwater and electricity. The generated electricity can then be used for electrolytic hydrogen production, establishing a sustainable integrated cycle.

The creative integration of i-TE conversion, high-efficiency thermally driven desalination, including revolutionary membrane-free, burgers cascade configurations, and water electrolysis has not only substantially enhanced individual unit performance but also established a robust technological foundation for high-performance, compact, and sustainable IWEH systems.

#### Smart building and continuous cycling

Smart buildings also offer a promising application domain for i-TE technology, leveraging temperature differences across building envelopes for distributed energy generation. Wei *et al*. [289] developed cement paste exhibiting i-TE effects with a thermopower of 1.06 mV K^−1^, facilitating integration into structural elements. Wu *et al*. [121] developed an i-TE paper chip that can power wireless alarms in fire safety systems. Fu *et al*. [290] designed thermosensitive ionic hydrogels for energy-harvesting smart windows, achieving an energy density of 250 mJ m^–2^. Waste heat from electronic devices can similarly be recovered to power sensors or small circuits, reducing dependence on external power sources.

Furthermore, i-TE materials can be further operated in a cycling mode, which has also been called thermally regenerated electrochemical systems, as a promising pathway for harvesting diurnal temperature variations. These systems leverage reversible electrochemical reactions whose thermodynamic potentials exhibit strong temperature dependence, enabling efficient conversion of the slow, day–night thermal cycles into continuous electrical energy [291]. Unlike conventional thermoelectric generators requiring sustained spatial temperature differences, cycling i-TE systems can store chemical energy during temperature swings, offering unique advantages for off-grid environmental monitoring and distributed power supply in remote areas.

## OUTLOOK AND ROADMAP FOR I-TE PRODUCTS

I-TE technology is demonstrably in a phase of rapid foundational development, with immense potential across emerging domains, such as wearable energy harvesting, self-powered sensing, and miniature-scale refrigeration [11,292]. However, it faces a formidable impediment in the current fragmented research landscape, including a lack of standardization in characterization, cross-scale design for innovative applications, scaling-up and reliability for commercialization, and critical thinking within the big picture of a sustainable and carbon-neutral endeavor.

### Standardization and specification of i-TEs

The standardization and specification are critical for any new rising field. Establishing a systematic, rigorous, and application-oriented standardization is desirable and an indispensable cornerstone for i-TE to realize its transformative application. This necessitates confronting two main challenges: pervasive ambiguity in fundamental definitions and terminology, and the lack of uniformity in methodologies for measuring key performance parameters.

The first significant challenge lies in terminology and definition, where disarray significantly hampers communication and the accumulation of knowledge. Chaotic naming conventions have accompanied the fast proliferation of novel i-TE electrolyte materials. Critical components, such as the polymer matrix, the specific active ions, and functional additives, frequently lack standardized descriptive norms. Establishing a common multi-dimensional material classification and naming standard is essential. This standard should systematically incorporate key defining characteristics, including the precise chemical composition, polymer type, crosslinker, solvent, and dominant microstructural features, such as porous, dense, and directional arrangement, the identity of the active ions governing the thermoelectric response, and critical physical states (for example, concentration). Concise descriptors, such as Aqueous-FeCN^4−/3−^-Gdm:Cl [38], Gelatin/GTA-KCl-FeCN^4−/3−^ [293], and PVDF-HFP/PEG-EMIM:TFSI-Na:TFSI [144], provide a practical template for achieving clarity and enabling unambiguous material identification and comparison across the research community. Furthermore, this ambiguity extends to the core metric of thermoelectric conversion efficiency, which is variably referred to as the Seebeck coefficient, or thermopower. This terminological fog creates substantial practical obstacles: it impedes efficient literature retrieval, hampers the reproducibility of research due to ambiguous descriptions, and fundamentally prevents effective cross-comparison of performance data from different groups, leading to stagnated knowledge accumulation. To navigate this confusion, a concerted effort toward terminological unification is paramount. An initial critical step is the broad adoption of a unified term, such as thermopower (*S*).

However, the challenge extends beyond nomenclature to the heart of experimental practice; the second critical bottleneck is the lack of uniformity in measurement solutions and conditions. This issue manifests acutely in determining thermopower. Significant, often uncontrolled, variations exist in nearly every aspect of the process: the method for applying and stabilizing the ${\mathrm{\Delta }}T$, the magnitude of the gradient, the degree of control over ambient temperature and humidity, and the timing of data acquisition after stabilizing ${\mathrm{\Delta }}T$. The cumulative effect is that ostensibly identical materials can yield vastly different reported thermopower values across laboratories, casting doubt on the reliability of the data. While thermopower originating from the thermogalvanic effect can be measured consistently using a stable three-electrode configuration, standardization is urgently needed for systems dominated by thermodiffusion. Common test configurations, such as the vertical method (where electrodes are vertical to the thermal gradient) and the coplanar method (where electrodes are parallel to the gradient), impose different thermal and ionic flow paths, potentially yielding different results and making direct comparisons invalid. Rigorous methodology requires that thermopower determination for a single sample involve continuous testing at multiple, sequentially applied temperature differences to generate a reliable ${\mathrm{\Delta }}T$ vs. ${\mathrm{\Delta }}V$ plot for linear regression. The impact of testing conditions is starkly illustrated by sample encapsulation. When tested unencapsulated, the measured voltage can be severely contaminated by moisture-electric effects, where voltage generation arises from humidity-induced water adsorption and desorption, or ion concentration gradients [294]. Consequently, the RH of the test environment can exert an enormous influence, with differences exceeding the order of magnitude (10-fold or more) reported between encapsulated and unencapsulated samples of the same material [295]. A critical gap is the absence of a universally trusted standard reference material specifically for characterizing thermodiffusion-dominated i-TE systems.

These inconsistencies extend to other critical properties. Electrical conductivity measurements employ various methods, including two-probe DC, four-probe DC, *I–V* characteristics, and EIS, yet often overlook the crucial contributions of electrodes and interfacial effects in devices. At the device level, performance assessment becomes even more complex. While the maximum instantaneous output power density (*P*_max_, derived from I–V curves under fixed ${\mathrm{\Delta }}T$) is helpful for rapid screening, it fails to capture the fundamental behavior of i-TE devices under sustained operation. A kinetic mismatch inevitably arises between the slow migration of ions within the electrolyte and the nearly instantaneous flow of electrons in the external circuit. This prevents maintaining a steady-state power output, causing the power to decay over time. Therefore, metrics such as thermoelectric conversion efficiency and the dimensionless figure of merit (*ZT*), calculated using instantaneous *P*_max_, are inapplicable and misleading for i-TE systems. A meaningful evaluation must assess the energy conversion efficiency over a complete discharge cycle, calculating the ratio of the total usable electrical energy delivered to the load to the total thermal energy input required to maintain the temperature gradient throughout the cycle. Developing a universal formula for this practical efficiency, alongside a corresponding meaningful figure of merit (*ZT*_i_) tailored to ionic dynamics, is an urgent and unmet need for the i-TE community.

These unresolved inconsistencies hinder accurate performance ranking, obscure optimization pathways, and undermine confidence in reported progress. Standardization should therefore be viewed not as a constraint on creativity, but as the essential foundation for maturing i-TE technology. Developing an open, dynamically evolving ecosystem—with clear nomenclature, standardized protocols, validated reference materials, and appropriate performance metrics—will enable reliable comparison and accelerate the transition from laboratory research to real-world applications.

### Cross-scale design for innovative application

The translation of i-TE technologies from laboratory prototypes to real-world applications demands a holistic, cross-scale design strategy that integrates material properties, device architecture, and system-level functionality. Such a comprehensive approach ensures not only high performance under controlled conditions but also robustness, scalability, and adaptability in diverse operational environments. This section outlines key considerations and emerging directions for innovative i-TE applications across the materials, devices, and system levels.

#### Materials-level engineering

**Figure 15.**
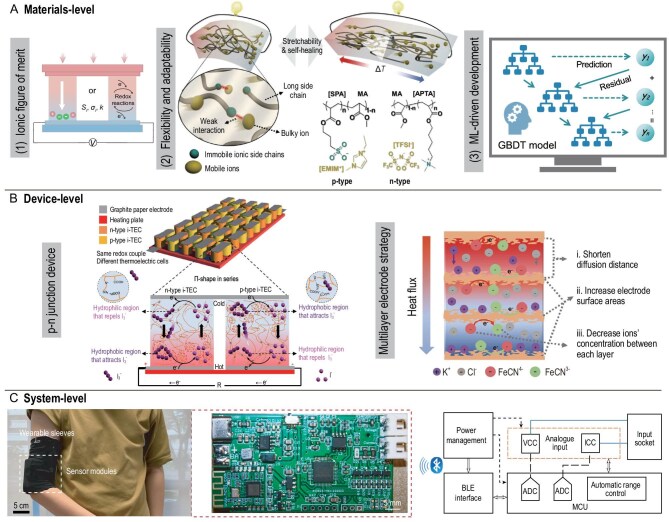
Representative cross-scale engineering strategies for i-TE applications. (A) Materials-level innovations targeting the ionic figure of merit, mechanical flexibility, and accelerated discovery via ML. Reproduced with permission from Ref. [298]. Copyright © 2023 Wiley-VCH GmbH. Reproduced from Ref. [323] under CC BY 4.0 license. Copyright © 2024 The Author(s), published by Oxford University Press on behalf of China Science Publishing & Media Ltd. (B) Device-level architectures featuring p–n junction thermopiles and multilayer electrodes for enhanced performance. Reproduced with permission from Ref. [154] under CC BY 4.0 license. Copyright © 2024 The Author(s). Reproduced with permission from Ref. [302]. Copyright © 2024 Wiley-VCH GmbH. (C) System-level integration for wearable applications, demonstrating embedded sensing, power management, and wireless communication. Reproduced with permission from Ref. [308]. Copyright © 2025 The Author(s), under exclusive licence to Springer Nature Limited.

At the material level, controlling heat and ion transport through the manipulation of micro- and nanostructures and ion–polymer interactions, is a widely adopted approach (Fig. 15A). The overarching goal is to decouple and tune the fundamental parameters governing i-TE performance, tailored to the requirements of targeted applications [172].

Energy harvesting: Effective material design should be integrated with thermal and electrochemical operating environments, ensuring that intrinsic thermopower translates into net power [296]. For the thermogalvanic components, it is crucial to select redox couples and electrodes that retain high reversibility and chemical cleanliness during thermal cycling, while utilizing quasi-solid or ionic gels to minimize convection and electrolyte leakage. For the thermodiffusion components, constructing a robust ion-selective asymmetry within the polymer network is necessary to generate substantial thermovoltages, while simultaneously enhancing ion diffusion rates without compromising mechanical stability. Furthermore, i-TE materials for heat harvesting must display balanced and robust properties for operation in real environments [297,298]. They should exhibit high ionic conductivity across the relevant temperature and humidity range but low thermal conductivity and minimal vapor loss to maintain an effective temperature gradient and operation stability.

Self-powered sensing: The large intrinsic thermopower of i-TE materials provides high sensitivity for detecting minute temperature variations, often with superior signal-to-noise ratios [299]. To enable high-frequency response and heat-flux sensing, i-TE materials must be engineered for high ionic mobility and minimized thermal mass. Long-term stability and environmental adaptability are also critical. Next-generation i-TE sensors must combine mechanical durability, environmental stability, and compatibility with multifunctional platforms to withstand complex and dynamic conditions. Ideal i-TE sensing materials should exhibit excellent flexibility, stretchability, and fatigue resistance to accommodate deformable surfaces and wearable systems, while maintaining consistent performance under varying humidity and temperature [300]. For biomedical and on-skin applications, additional requirements include biocompatibility, use of non-toxic components, and long-term stability under physiological fluctuations [247].

Moving i-TE materials toward real-world applications requires a shift beyond the conventional ionic figure of merit. True application readiness hinges on a combination of mechanical robustness, environmental durability, and ease of integration. The vast compositional complexity of i-TE platforms makes empirical optimization challenging. ML offers a powerful means to accelerate material discovery and development. For example, gradient boosting decision tree models can identify key molecular features that most strongly influence i-TE performance, such as the number of rotatable bonds and octanol–water partition coefficients of ions, thereby guiding the design of new material systems with optimized properties [284]. Future ML frameworks have the potential to perform multi-objective optimization, balancing thermopower, ionic conductivity, toughness, and environmental stability (Fig. 15A).

#### Device-scale innovation

Realizing high-performance i-TE devices requires a co-design approach that integrates both thermal and electrochemical considerations at the device level. Two principal design architectures are commonly employed: lateral (in-plane) configurations and vertical (through-thickness) stacks [301]. Lateral architectures situate electrodes on the same plane and enable facile patterning and integration with textiles. However, lateral heat spreading can reduce the effective temperature gradient across the device. In contrast, vertical stacks align the thermal and electrochemical pathways through the device thickness, more effectively matching the skin-to-ambient heat flow and aiding in retention of Δ*T* under deformation. Device design should be treated as a thermal-resistance network. This involves integrating low-impedance thermal interface materials on the hot side, coupling the cold side with efficient heat-exchange structures, and minimizing dehydration through the use of anti-drying ionogels or organo-hydrogels [134].

Optimizing device-scale performance necessitates the simultaneous maximization of key parameters, including power density, energy density, Carnot-relative energy conversion efficiency, and sustained power generation under fixed thermal boundary conditions [11]. One pivotal strategy to enhance output voltage and power is the construction of p–n junction devices, wherein p-type and n-type i-TE legs are connected in series to form thermopiles (Fig. 15B) [154]. This architecture scales output voltage multiplicatively, which is essential for powering practical electronics, but also requires precise matching of the electrical, ionic, and mechanical properties between two materials to minimize internal losses. Time-constant engineering should also be considered to minimize the thermal charging time, ensuring that high intrinsic thermopower translates into usable power. Recently, the implementation of multilayer and 3D electrode designs has proven effective in shortening ion-diffusion lengths and reducing internal resistance, dramatically decreasing the thermal charging time and boosting both instantaneous power and sustained energy output (Fig. 15B) [302]. In addition, the interfacial EDL serves as a fundamental regulator of charge-transfer resistance and the structural integrity of interfaces during sustained thermal cycling. By strategically modulating functional group density and chain orientation to facilitate ion-solvation-mediated hopping, while integrating high-surface-porosity architectures to ensure rapid transport kinetics, a synergistic electrode paradigm is established [303]. This functional synergy is further amplified by multi-scale architectural engineering [304], wherein the integration of 3D hierarchical frameworks [305,306] and vertically aligned porous channels [307] achieves the co-optimization of electrochemically accessible surface area and ion transport kinetics.

Reliability and robust packaging are crucial for real-world deployment. Essential design features include stable, low-impedance electrode/electrolyte interfaces, chemically durable electrodes and interconnects, vapor-tight encapsulation to limit water loss, and anti-drying electrolytes to maintain performance during motion, humidity fluctuations, and long-term operation.

Future priorities at the device scale include standardized performance benchmarking under realistic thermal contacts, scalable fabrication of multilayer electrodes to further reduce charging time, packaging strategies validated for mechanical and environmental durability, and the development of electrolyte formulations that sustain high ionic mobility and stability over broad temperature and humidity ranges [14].

#### System-level engineering

At the system level, the challenge is to seamlessly integrate i-TE devices with complementary technologies into robust, application-ready platforms (Fig. 15C). A promising frontier lies in the development of multi-physical field coupled systems that synergistically harness thermal energy alongside other ambient energy sources. By integrating i-TE components with mechanisms for capturing light, magnetic fields, or moisture variations, these hybrid systems can achieve significantly enhanced energy harvesting efficiency and functional robustness in real-world environments. The future development of i-TE systems should capitalize on their unique attributes to enable multifunctional capabilities. The integrated platforms can support self-powered sensing with on-board signal processing, hybrid energy harvesting that shares heat spreaders with photovoltaics or conventional thermoelectrics, and building panels that combine energy buffering with precise heat mapping. Realizing these functions requires outstanding system compatibility, which entails maintaining stable electrochemical performance under mechanical stress (bending, vibration), regulating hydration for prolonged duty cycles, ensuring biocompatibility for skin-contact applications, and implementing benign thermal interfaces for interaction with human or architectural substrates [308].

The design of these multi-physical field systems necessitates a holistic approach that considers the interplay between different energy conversion mechanisms. For instance, Janus-structured films with asymmetric optical and thermal properties can simultaneously manage solar absorption and radiative cooling, creating sustained temperature differences for i-TE generation. Similarly, cellulose-based composites with aligned nanochannels can couple moisture-induced potential with thermal voltage, enabling all-weather energy harvesting. These advanced material architectures underscore the importance of cross-coupling effects in the design of next-generation i-TE systems.

Transitioning from proof-of-concept to practical deployment demands a closed-loop development workflow, coupling materials screening, device prototyping, system simulation, and field validation. Within this framework, Multiphysics models that bridge heat transfer, ion transport, and charge storage can be matched with uncertainty-aware optimization, ML, and high-throughput experimentation to optimize geometry, electrolyte composition, and operational protocols simultaneously. For multi-physical field systems, these models must be extended to incorporate additional physical processes such as photothermal and hygrothermal dynamics, enabling accurate prediction of system behavior under complex environmental conditions. Concurrently, scenario-driven prototyping under realistic boundary conditions delivers essential feedback to guide iterative refinement of materials and packaging strategies. Sustainable, scalable manufacturing should be embedded in the design process from the outset, favoring roll-to-roll or printable fabrication, solvent-lean chemistries, the use of abundant and non-toxic materials, recyclable electrodes and housings, and life-cycle assessments to guide materials selection.

A representative example of this approach is a portable TGC-based dressing system for smart wound monitoring and analysis (Fig. 15C) [308]. In this system, a Fe^2^⁺/Fe^3^⁺ redox-coupled alginate hydrogel thermocell is integrated with a flexible circuit board supporting Bluetooth Low Energy (BLE) communication, analog-to-digital conversion (ADC), and power management. This platform enables fully self-powered monitoring of wound temperature, detection of exudate levels via impedance measurements, and respiratory rate assessment, while also supplying real-time electrical stimulation to promote healing. By uniting energy harvesting, biosensing, and therapeutic intervention, this system exemplifies multifunctional integration at the system level.

Beyond the immediate challenges of material synthesis and device integration, a successful ‘ions-to-products’ translation necessitates a fundamental reconciliation with the transient, non-steady-state dynamics intrinsic to i-TE power delivery. Unlike conventional electronic thermoelectric generators that provide a constant DC voltage under a steady temperature gradient, i-TE devices—particularly those utilizing the thermodiffusion effect—typically produce transient, pulsed, or exponentially decaying power profiles. This characteristic creates a significant functional gap when interfacing with standard microelectronic loads. To bridge this gap, the development of customized power management systems or power management integrated circuits is indispensable [309]. Such systems must incorporate adaptive impedance matching to compensate for the dynamic fluctuations in internal resistance as ionic concentration gradients evolve and relax. Furthermore, specialized maximum power point tracking algorithms [310,311], designed for ultra-low frequency and non-steady-state sources, are required to optimize energy extraction efficiency across varying duty cycles. By integrating i-TE modules with energy-buffering components—such as low-leakage supercapacitors or thin-film batteries—and customized voltage-boost converters, the intermittent ionic signals can be regulated into a stable power supply with continuous effort. This system-level integration is essential for enabling i-TE technology to drive autonomous sensors and communication modules within the broader IoT and wearable ecosystems.

Looking ahead, the advancement of i-TE products will depend on moving beyond single performance metrics and embracing holistic, cross-scale engineering. Key future directions include: (i) AI-driven discovery of next-generation i-TE materials with tailored responses to multiple physical stimuli; (ii) advanced fabrication of complex device architectures tailored for optimized multi-physical field coupling and thermal pathways; and (iii) development of standardized, plug-and-play i-TE modules for seamless integration into broader platforms such as IoT devices, smart wearables, and industrial energy management systems. Particular emphasis should be placed on creating systems that can adaptively harness diverse environmental energies based on availability, significantly expanding the operational scenarios and energy output stability of i-TE technologies. Through coordinated design efforts spanning from molecular engineering to complete system integration, the path toward widespread, impactful adoption of i-TE technology becomes both straightforward and achievable.

### Sustainable and carbon-neutral endeavor

#### Sustainable and recyclable materials

The adoption of sustainable and recyclable materials represents two complementary strategies for enhancing the sustainability of i-TE systems. The former emphasizes the use of environmentally friendly raw materials and low-impact manufacturing processes, while the latter focuses on end-of-life recyclability. Their integration can substantially reduce the carbon footprint and environmental impact across the entire lifecycle of i-TE materials. A typical i-TE device comprises electrodes, an electrolyte, and packaging materials, with electrolytes receiving the most intensive research focus. Conventional redox couples, such as Fe^2+/3+^, [Fe(CN)_6_]^4−/3−^, and I^−^/I_3_^−^, are predominantly employed in aqueous electrolytes. However, recent trends indicate a shift toward organic solvent-based systems, which enhance configurational entropy and gel-based electrolytes, offering superior stability and mechanical flexibility. The key advances include the use of natural polymers (for example, cellulose, chitosan, and gelatin) and eco-friendly solvents such as ILs or aqueous systems, highlighting the potential of green chemistry in i-TE design.

Recent breakthroughs demonstrate the viability of bio-based and recyclable materials for high-performance i-TE systems. Li *et al*. [119] pioneered fibrillated cellulose as a petroleum alternative, achieving superior mechanical strength, recyclability, and biodegradability through aligned ion-transport channels. Feng *et al*. [96] further extended this approach by combining BC with ILs, yielding robust ionogels. Similarly, Han *et al*. [4] utilized waste-sourced gelatin, while Sun *et al*. [312] developed biomimetic chitosan-based hydrogels with laminated porous structures via freeze-casting, enhancing both performance and sustainability.

Recyclability is another critical aspect of i-TE material design, which directly addresses end-of-life challenges. Zhao *et al*. [180] developed PEO-LiTFSI-EMIM:Cl ionogels featuring dynamic Li⁺–O coordination and a hydrogen-bonding network. These interactions permit mild ethanol-assisted disassembly, molecular-level separation, and closed-loop reassembly via solvent evaporation. This end-of-life strategy enables material regeneration with minimal performance loss (for example, <2% drop in thermopower after recycling), offering a practical route to sustainability. Jheng *et al*. [313] developed a double-network ionogel using α-lipoic acid, hydroxypropyl cellulose, and acrylic acid. The material exploits reversible disulfide bonds and hydrogen bonding to enable complete melting at 165°C and re-molding upon cooling, achieving solvent-free recycling. The recycled gel maintained an n-type thermopower of −11.0 mV K^−1^, confirming excellent reusability without performance degradation.

Clearly, these studies highlight the potential of natural and bio-inspired polymers for designing high-performance and eco-friendly i-TE materials, suggesting a future where sustainable design is seamlessly integrated with advanced thermoelectric properties. To translate this potential into practical applications, further research should focus on expanding green material sources and developing scalable fabrication techniques. Notably, the incorporation of renewable feedstocks with reversible bonding chemistries offers a promising strategy for creating i-TE materials that remain sustainable throughout their entire lifecycle, which is from production to disposal. In parallel, comprehensive life-cycle assessments will be essential to quantitatively evaluate environmental benefits without compromising thermoelectric performance.

#### Device lifespan extension

In addition to sustainable and recyclable materials, extending the operational lifespan of i-TE devices is essential for reducing resource consumption. However, most i-TE systems remain confined to laboratory-scale demonstrations, with research often prioritizing high thermopower over long-term stability. Aqueous electrolytes, for instance, face challenges such as solvent evaporation and poor sealing, whereas hydrogels tend to dehydrate over time at room temperature, leading to performance degradation. Emerging strategies to improve device longevity include humidity management, advanced encapsulation, and self-healing mechanisms.

Humidity-control approaches aim to minimize water loss by enhancing moisture retention or utilizing solid ionogels. Zhang *et al*. [314] developed a ternary PEDOT:PSS/PVA/polyacrylate hydrogel with exceptional water retention, enabling a reversible dehydration-rehydration cycle that restored 87% of its original performance after 135 days. Similarly, Li *et al*. [315] introduced a self-hydrating system using glycerol and hygroscopic salts, which autonomously replenishes water from ambient air, achieving a thermopower of 4.1 mV K^−1^ and extending continuous operation to 1500 min. For solid ionogels, Feng *et al*. [316] designed a hygroscopic ionogel that boosts voltage output 2–3 times and extends operational time beyond 12 h. Zhao *et al*. [95] reported a stretchable ionogel maintaining over 80% performance after 600 h with a thermopower of 18 mV K^−1^. Jia *et al*. [107] demonstrated a DES-based gel that retained >90% water content and showed no performance loss over 31 days. Encapsulation strategy such as using polyvinylpyrrolidone (PVP) to protect I^−^/I_3_^−^ systems from iodine volatilization was proved to be sustainable over hundreds of thermal cycles. Moreover, self-healing capabilities also contribute significantly to durability. For example, Kim *et al*. [114] developed an intrinsically self-healing proton gel, capable of autonomous repair within 60 s. Alternatively, Malik *et al*. [317] used SiO_2_ nanoparticles to enable extrinsic self-healing via hydrogen bonding within 2 min. Despite these localized advances, pervasive challenges such as redox couple degradation, metal-electrolyte interfacial corrosion, and the maintenance of robust interfaces against ion penetration through encapsulation materials remain critical hurdles that warrant more intensive investigation. Furthermore, moving toward industrial translation, scalable fabrication and comprehensive life-cycle assessments are essential for synergizing high thermoelectric performance with long-term environmental sustainability.

### Scaling-up and reliability for commercialization

The widespread adoption of intelligent i-TE products depends critically on achieving scalable manufacturing and ensuring long-term device reliability. Translating promising lab-scale demonstrations into practical products requires overcoming barriers in cost, yield, and reproducibility. Standardized, high-throughput fabrication methods—such as roll-to-roll processing, additive manufacturing, and automated packaging—are essential to reduce production costs while maintaining performance uniformity. Integrating real-time quality monitoring and defect control into scalable protocols will further accelerate the shift from prototypes to volume production. Equally crucial is demonstrating reliability under realistic operating conditions, including thermal cycling, mechanical deformation, and environmental variations. Systematic evaluation—through accelerated lifetime testing, interfacial stability analysis, and encapsulation optimization—must be embedded in the development process. Cross-disciplinary collaboration will be key to building comprehensive reliability databases that inform material selection and engineering design, ultimately guiding i-TE technology toward successful commercialization.

For the widespread adoption of i-TE technology, cost-effectiveness is as crucial as performance, defined as achieving target performance at minimal lifetime cost. While i-TE devices typically outperform traditional thermoelectrics in sensitivity due to their giant thermopower, their slow ionic diffusion limits response speed, making them suitable for applications without stringent time constraints (for example, gas sensing and heat flux measurement). In terms of cost, i-TE devices hold advantages in material expenses, as polymer-based electrolytes are cheaper and can be processed via low-cost solution methods (for example, printing and coating) onto diverse substrates. However, challenges remain in micro-scale fabrication due to incompatibility with standard microelectronic processes and in forming reliable electrode connections—issues that strategies such as developing all-solid-state ionic materials or conductive polymer interfaces aim to address but still pose bottlenecks for large-scale manufacturing [137].

### CONCLUSION

In summary, we have given a comprehensive review of the i-TE field, with a bird’s-eye view on the recent progresses in materials design, device construction, and novel applications. The i-TE field is still in its infancy, but is growing quickly. It has a unique complexity from the microscopic mechanisms to the macroscopic applications, attracting attentions of materials scientists, chemists, physicists, and engineers. It shows many innovative applications in wearable scenarios, including but not limited to stretchable power generation from body heat, thermal sensing, and intelligent human–machine interactions. It also shines light on dealing with global environmental challenges, such as IWEH generations and smart buildings. These engineering advances might need more cooperation from multidisciplinary fields, to overcome the challenges of standardization, scalable manufacturing and reliability, and to boost their economic value.
